# Resistance development in *Escherichia coli* to delafloxacin at pHs 6.0 and 7.3 compared to ciprofloxacin

**DOI:** 10.1128/aac.01625-22

**Published:** 2023-10-26

**Authors:** Anja Bösch, Magreth E. Macha, Qun Ren, Philipp Kohler, Weihong Qi, Baharak Babouee Flury

**Affiliations:** 1 Medical Research Center, Kantonsspital St. Gallen, St. Gallen, Switzerland; 2 Division of Infectious Diseases and Hospital Epidemiology, Kantonsspital St. Gallen, St. Gallen, Switzerland; 3 St. Francis University College of Health and Allied Sciences, Morogoro, Tanzania; 4 Laboratory for Biointerfaces, Empa, Swiss Federal Laboratories for Materials Science and Technology, St. Gallen, Switzerland; 5 Functional Genomics Center Zurich, University of Zurich, ETH Zurich, Zurich, Switzerland; University of Pittsburgh, Pittsburgh, Pennsylvania, USA

**Keywords:** delafloxacin, *Escherichia coli*, ciprofloxacin, resistance evolution, pH

## Abstract

Understanding the resistance mechanisms of antibiotics in the micro-environment of the infection is important to assess their clinical applicability and potentially prevent resistance development. We compared the laboratory resistance evolution of *Escherichia coli* to delafloxacin (DLX) compared to ciprofloxacin (CIP), the co-resistance evolution, and underlying resistance mechanisms at different pHs. Three clones from each of the eight clinical *E. coli* isolates were subjected to subinhibitory concentrations of DLX or CIP in parallel at either pH 7.3 or 6.0. Minimum inhibitory concentrations (MICs) were regularly tested (at respective pHs), and the antibiotic concentration was adjusted accordingly. After 30 passages, MICs were determined in the presence of the efflux pump inhibitor phenylalanine-arginine-β-naphthylamide. Whole genome sequencing of the parental isolates and their resistant derivatives (*n* = 54) was performed. Complementation assays were carried out for selected mutations. Quantitative PCR and efflux experiments were carried out for selected derivatives. For DLX-challenged strains, resistance to DLX evolved much slower in acidic than in neutral pH, whereas for CIP-challenged strains, the opposite was the case. Mutations in the quinolone resistance-determining region were mainly seen in CIP-challenged *E. coli*, whereas a multifactorial mechanism including mutations in efflux-related genes played a role in DLX resistance evolution (predominantly at pH 6.0). This work provides novel insights into the resistance mechanisms of *E. coli* to delafloxacin and highlights the importance of understanding micro-environmental conditions at the infection site that might affect the true clinical efficacy of antibiotics and challenges our current antibiotic susceptibility-testing paradigm.

## INTRODUCTION

Delafloxacin (DLX), a novel dual-targeting nonzwitterionic fluoroquinolone, has received approval by the US Food and Drug Administration (FDA), the European Medicines Agency (EMA), and Swissmedic for the treatment of acute bacterial skin and skin structure infections and community-acquired bacterial pneumonia in adults (FDA and EMA), caused by designated susceptible bacteria. DLX has excellent activity against Gram-positive organisms and anaerobes and similar minimum inhibitory concentrations (MICs) to those of ciprofloxacin (CIP) against Gram-negative bacteria, with MIC_90_ of 0.06, 0.5, and 0.25 mg/L for *Escherichia coli*, *Klebsiella pneumoniae*, and *Enterobacter* spp., respectively (1). It has anionic character at neutral pH (~7–7.4) and is mainly found in uncharged form at slightly acidic pH, which differs from other fluoroquinolones (e.g., CIP, levofloxacin, moxifloxacin) that are present as cations at acidic pH and mainly zwitterions at higher values, and for which activities decrease (higher MICs) in acidic environments (2 – 4). This feature may explain the highly improved potency of DLX in acidic environments, as the nonionized form is considered more diffusible through biological membranes (5).

Standard *in vitro* antibiotic susceptibility testing conditions do not always reflect the micro-environmental conditions of the infection site. Urines of patients with suspected *E. coli* or *K. pneumoniae* urinary tract infections exhibit pH 6.5 or less (6); the same applies for biofilms, including those involving *E. coli*, where pH ranges between 3.5 and 6.0 have been documented (7 – 10); the pH of purulent abscess fluids ranges from 5.5 to 7.2 (11, 12). There is only very little literature available on the range of pH in intraabdominal abscesses, which has been documented to be 5.9–7.2 (13), 6.75 in median (12), and 5.5–6.8 (14).

Data on the effectiveness of DLX in Gram-negative pathogens, including *E. coli*, as well as their resistance mechanisms against DLX, are still scarce. This study sought to determine the resistance- and cross-resistance evolution of *E. coli* against DLX and CIP at pHs 6.0 and 7.3 and to decipher the resistance mechanisms against DLX.

## RESULTS

### Multistep resistance selection and determination of minimum bactericidal concentration

**FIG 1:**
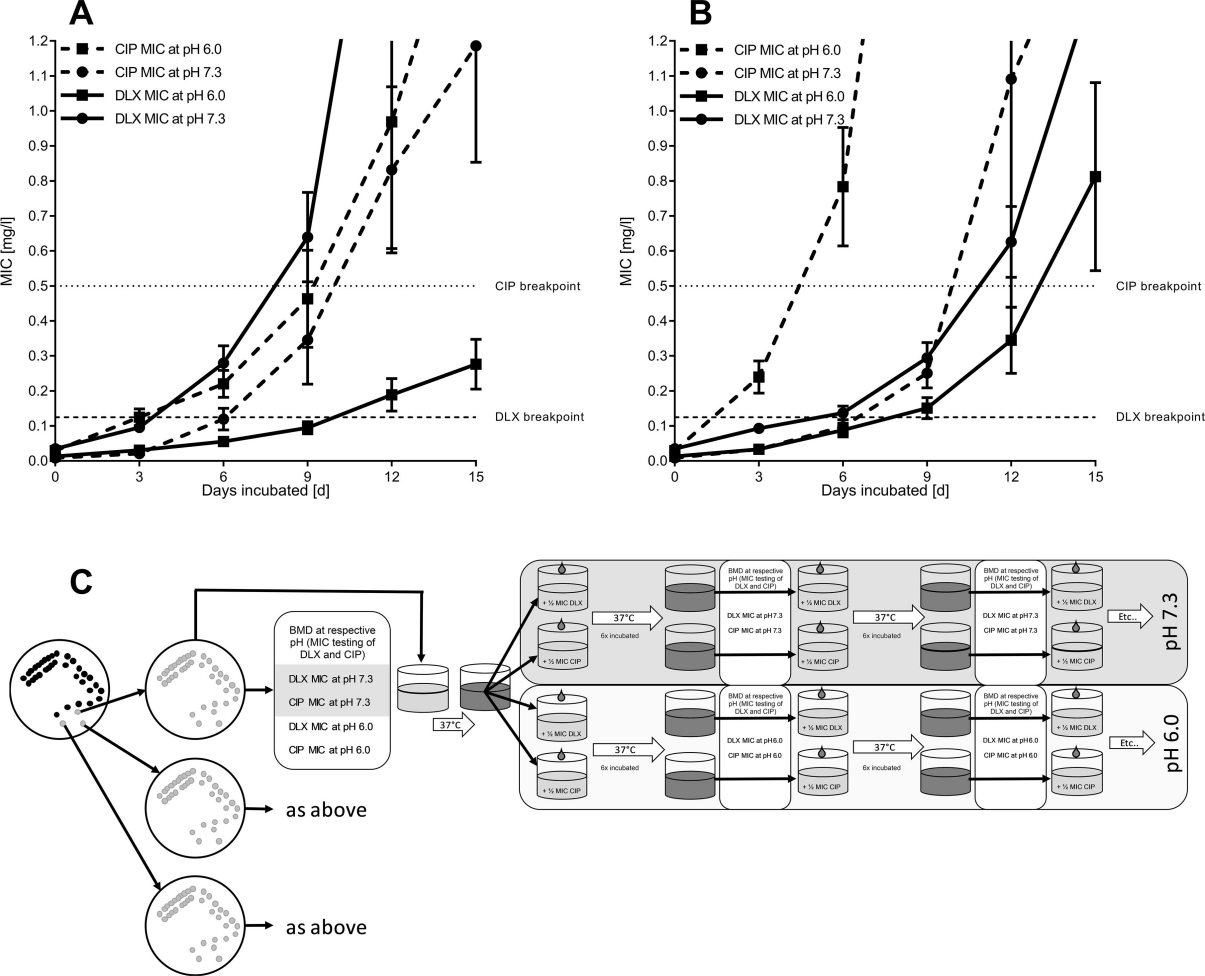
Resistance evolution of *E. coli* passaged in subinhibitory concentrations of delafloxacin or ciprofloxacin at pHs 7.3 and 6.0. (**A**) *E. coli* incubated in subinhibitory (1/2 MIC) concentrations of DLX. (**B**) *E. coli* incubated in subinhibitory (1/2 MIC) concentrations of CIP. Depicted are the mean values and the standard error of the mean (SEM) of the isolates passaged. EUCAST breakpoints are shown as a dotted line at *y* = 0.5 mg/L for CIP and a dashed line at *y* = 0.125 mg/L for DLX. Solid lines represent DLX-MICs, and dashed lines represent CIP-MICs. Circles show the MICs at pH 7.3, and squares show the MICs measured at pH 6.0. (**C**) The MICs for DLX and CIP at different pHs (pH 6.0 or 7.3) were determined for three clones of an isolate via the broth microdilution method. Afterward, the isolates were alternately cultured six times in subinhibitory antibiotic concentrations at respective pHs over 3 days, with an adjustment of the pH twice daily. After these passages, the MIC was redetermined to adjust the antibiotic concentration for the next incubation step.

In order to collect resistant derivatives for further molecular investigations, the clones were subjected to subinhibitory concentrations of respective antibiotics (i.e., 1/2 of the MIC tested), until at least one clone from each of the parental isolates had surpassed the resistance-breakpoint according to the European Committee on Antimicrobial Susceptibility Testing (EUCAST) (Fig. 1A and B) (15). Therefore, the clones were cultivated for 15 days, which comprised 30 passages in total.

Resistance evolution for DLX at pH 6.0 was substantially slower than that at pH 7.3, whereas at pH 7.3, only 3/24 clones incubated in subinhibitory concentrations of DLX were in the susceptible range after 12 days, and all clones had acquired DLX resistance (MIC >0.125 mg/L) after 15 days. On the other hand, only 9/24 clones (37.5%; derived from five isolates) (*P* < 0.001) had reached an MIC >0.125 mg/L at pH 6.0 (see Fig. S1). An opposing trend was observed for CIP resistance evolution after being passaged in subinhibitory concentrations of CIP; 87,5% (21/24 clones) developed resistance at pH 6.0, whereas at pH 7.3, MICs of 46% (11/24 clones, derived from six isolates) rose above 0.5 mg/L (*P* = 0.002) (Fig. S1). Cross-resistance to DLX occurred in 83.3% (20/24) of the clones passaged in subinhibitory concentrations of CIP at pH 7.3 and in 62.5% (15/24) of the clones at pH 6.0, respectively (*P* = 0.10). Cross-resistance development to CIP was observed in 37.5% (9/24) and 41.6% (10/24) clones challenged to subinhibitory concentrations of DLX at pHs 7.3 and 6.0, respectively (*P* = 0.77). In total, the development of DLX-cross-resistance in strains challenged with CIP was significantly more common than that of CIP-cross-resistance in those challenged with DLX, 72.9% (35/48) vs 39.9% (19/48), respectively (*P* < 0.001). The detailed MIC evolution of single clones from each parental isolate is depicted in Fig. S2 (supplemental material). Untreated controls did not exhibit a rise in MIC outside a range of ±0.01 mg/L (Fig. S3, supplemental material). Detailed information on MICs for several additional antibiotic compounds tested against the derivatives is depicted in Table S1. Of note, we did also observe raised MICs for aminoglycosides at pH 6.0 (16).

In addition to MIC, minimum bactericidal concentration (MBC) was determined for the final derivatives on day 15 of passaging for DLX and CIP. MBCs were identical to MICs for 95% (91/96) of all derivatives tested. For the five derivatives that did not harbor identical MBC and MIC, the MBC/MIC ratio was 2 for DLX in two derivatives and 4 for CIP in three derivatives, showing that the mutations acquired are insufficient by themselves to abolish the bactericidal activity of fluoroquinolones *in vitro*.

### Efflux pump inhibition assay

**FIG 2:**
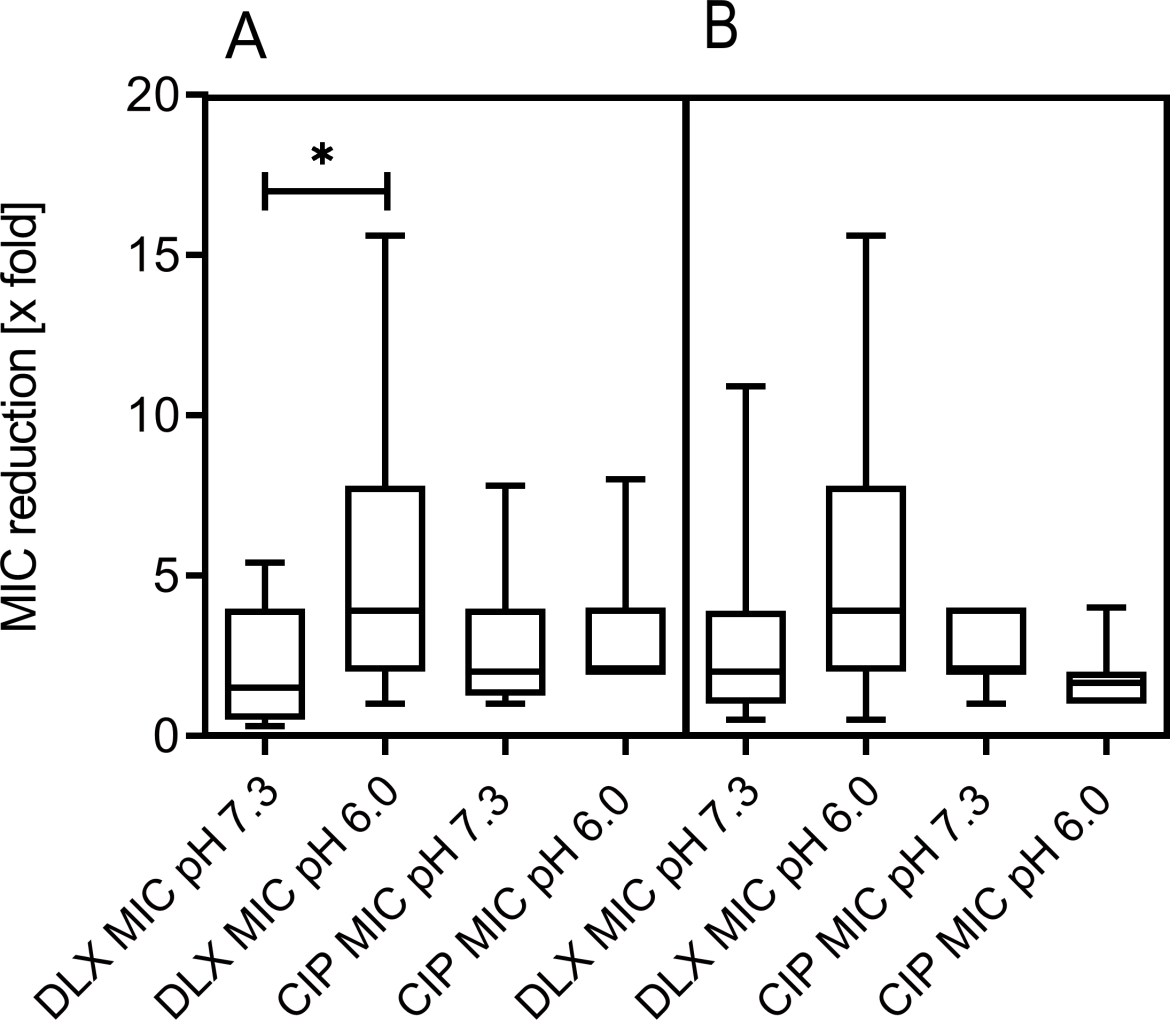
Ratio of MIC reduction in resistant derivatives compared to parental isolates after efflux pump inhibition by PaβN. (**A**) *E. coli* incubated in subinhibitory (1/2 MIC) concentrations of DLX. (**B**) *E. coli* incubated in subinhibitory (1/2 MIC) concentrations of CIP. *, *P* < 0.05; *y*-axis, MIC reduction (*x*-fold) calculated as a ratio of MIC reduction in derivatives divided by MIC reduction in parental isolates after addition of PaβN; *x*-axis, MICs of the respective antibiotics and pHs (6.0 vs 7.3) the bacteria were subjected to.

The MIC reduction fold for the efflux pump inhibition assay was calculated as a ratio of MIC reduction in resistant derivatives divided by MIC reduction in parental isolates after the addition of phenylalanine-arginine-β-naphthylamide (PaβN), in order to take into account how PAβN affects the MIC in both parental strains and their resistant derivatives. Efflux pump inhibition with PaβN leads to a significant reduction of the DLX-MIC in clones passaged at pH 6.0 compared to those passaged at pH 7.3 (Fig. 2; Fig. S4). In contrast, efflux pump inhibition did not essentially change the MICs for CIP (mean twofold).

### Quantitative PCR

**FIG 3:**
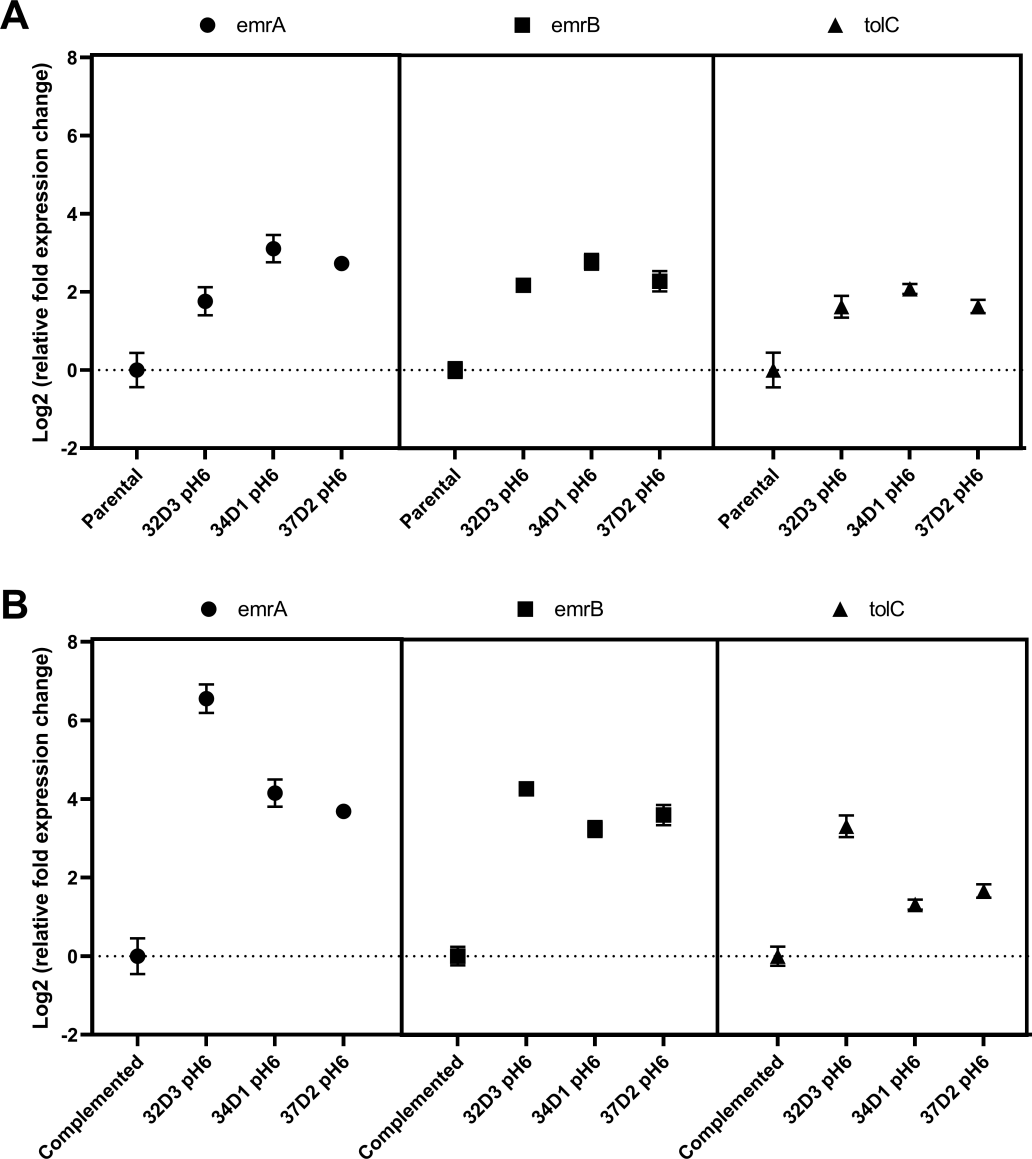
Gene expression level changes for genes *emrA*, *emrB*, and *tolC* in delafloxacin-resistant mutants. All expression levels were log_2_ transformed and normalized to expression levels observed in the parental (**A**) or complemented strains (**B**). Error bars represent the standard deviation (SD). For reference strains, means of SDs are depicted.

A quantitative PCR (qPCR) analysis of *emrAB* and *tolC* genes was performed to examine the role of their expression on delafloxacin resistance (Fig. 3). For this purpose, we examined the three derivatives, for which complementation of the *wt-emrR* gene exhibited the highest DLX-MIC-fold reduction (Table 2), i.e., 32D3, 34D1, and 37D2, all cultured at pH 6, exhibiting an MIC-fold reduction of 8-, 4-, and 5.4-fold, respectively. A twofold or more expression level change was considered to be biologically significant (17).

In the derivatives 32D3, 34D1, and 37D2, compared to their respective parental isolates, we observed a 3.4-, 8.6-, and 6.6-fold and a 4.5-, 6.8-, and 4.8-fold increase of the *emrA* and *emrB* and a 3-, 4.2-, and 3-fold increase in *tolC* expression, respectively.

We also compared the expression levels of *emrAB* and *tolC* of the derivatives compared to their complemented counterparts. Here, we observed a 94-, 17-, and 13-fold and a 19-, 9-, and 12-fold increase of the *emrA* and *emrB* and a 10-, 2.4-, and 3-fold increase in *tolC* expression, respectively, suggesting an additional repression of *emrAB-tolC* through the introduced repressor gene.

### Whole genome sequencing

Whole genome sequencing was performed on the parental isolates and selected resistant derivatives. From each parental isolate, at least one resistant derivative at the end of the multistep resistance selection was chosen for further characterization by whole genome sequencing (WGS). Additional derivatives were characterized to see if they differed in their MIC evolution compared to the other clones of the same parent. A complete list of mutations detected between parental isolates and their selected resistant derivatives is shown in Table S3.

Mutations in genes coding for topoisomerase enzymes and efflux-related genes are summarized in Table 1. Mutations in *gyrA* were seen in all derivatives that had been challenged with CIP, regardless of the pH the strains were incubated at. They all comprised mutations at the S83 and D87 sites, which are two mutational hotspots for quinolone resistance located on the GyrA helix-4(18).

**TABLE 1:** Selected mutations in topoisomerase- and efflux-related genes^*a*^

			Topoisomerases				Efflux-related genes							
		Derivative ID	gyrA	gyrB	parC	parE	emrB	mdtM	emrK	emrR	marR	mdtC	acrR	soxR
**Incubation in subinhibitory concentrations of DLX**	**At pH 6.0**	31D1								**R110H**	**G69R**			
		31D3	R612L								**R73C**			
		32D2	T719N								**R73C;** **M74G**			
		32D3	S83L		**R564S**					**S84Y**				
		33D3	R612G											
		34D1	**G616S**							**R111C**	**G69E**	**V632I**		
		34D3	**S83L**			R611C				**Q138K**		**V632I**		
		36D2	S83L			R611L								
		37D2								**D109A**	**T72P**			
		39D2								**R111S**				
		42D3												
	**At pH 7.3**	31D1	T611I											
		31D3	**A614V**							**L159P**	**L78P**			
		32D2												
		32D3	S83L			P398L				**W140R**	**T39A**			
		33D1	S83L		T140A							P183A		I59V, A146V
		34D3	**D87G; S83L**		A117V					**L151H**		**V632I**		
		36D1		A315V						Q150K				
		36D2	**S83L**		**A117V**					**D109G**				
		37D2	S83L			P398L					**T72P**			
		39D3												
		42D1	Y605C							L64P				
**Incubation in subinhibitory concentrations of CIP**	**At pH 6.0**	31C2	D87G	S464F	A117E									R20C
		31C3	S83L								**E85I;** **R86S;** **L87W**			
		32C2	D87G	S464Y		E460K								
		32C3	S83L											
		33C3	D87G;S83L		G78C	S458W								
		34C2	D87A											
		34C3	S83L									**V632I**		
		36C3	D87Y											
		37C1	D87Y; S83L		S80R	D420G								
		37C3	D87Y								T72P			
		39C3	D87G			I444F								
		42C2	D87N											
	**At pH 7.3**	31C3	D87G;S83L											
		32C1	D87G				I152D; I156N						W178R	
		32C2	D87G											G121D
		33C1	D87G		R616C; S80I			T98A	G273D					
		33C3	D87G										E142D	
		34C1	D87Y								**G69E**	V632I		
		34C2	D87Y							**T56A;** **F58I**		V632I		
		36C3	D87G										V29G	
		37C3	D87Y											
		39C3	D87G											
		42C2	S83L											

^*a*^ CIP: ciprofloxacin; DLX delafloxacin. Mutations are represented as amino acid changes (one letter code); bold, complemented.

For strains challenged with DLX, S83L was the predominant mutation seen in sequenced derivatives (8/22); however, not all *gyrA* displayed mutations, and 7/22 showed mutations outside the conserved regions known as the quinolone resistance-determining region (QRDR) (19). Mutations in *gyrB* were far less frequently encountered.

Regarding efflux-related genes, the main differences between CIP- and DLX-challenged strains were mutations detected in *emrR*, which were predominantly seen in DLX-challenged clones (i.e., 2 vs 12 mutations, respectively) (Table 1). For those strains challenged with CIP, mutations in efflux-related genes were more common at pH 6.0 than at pH 7.3 (12 vs 5). *MarR* and *mdtC* mutations were seen in both CIP- and DLX-challenged strains, with V632I being the most common mutation in *mdtC* for both DLX- and CIP-challenged strains. Mutations in *emrB*, *mdtM*, *emrK*, and *acrR* were exclusively detected in a few CIP-challenged strains.

From the selected derivatives, we retrospectively also sent the clones from days 9 (passage 18) and 12 (passage 24) for WGS in order to examine for recurrent or random mutations in the time between. For all protein-coding genes, we calculated the ratio of the number of nonsynonymous substitutions per nonsynonymous site to the number of synonymous substitutions per synonymous site (i.e., dN/dS) using SNPGenie (20). The efflux-related genes and the topoisomerase genes reported in our study had higher dN/dS compared to the other genes. The dN/dS around 1 and greater suggests that the reported genes identified in our study are likely under a strict neutral evolution in nonsynonymous substitutions and/or positive selection (Fig. S5). Finally, for each derivative, the total number of missense single nucleotide polymorphisms (SNPs) in specific genes was counted and plotted against time, showing the accumulation of missense SNPs over time (Fig. S6).

### Complementation assays (Tables 1 and 2)

Complementation was performed for all mutations found in efflux-related genes in DLX-challenged, resistant derivatives that were incubated at pH 6.0 listed in Table 1. The reason for the choice of specific efflux-related genes for complementation was the results from the efflux pump inhibition with PaβN, highlighting a significant reduction of the DLX-MIC in clones passaged at pH 6.0 compared to those passaged at pH 7.3 and CIP. We succeeded in introducing the respective wild-type (wt) gene in all these derivatives, except for *mdtC* into 33D1, as the derivative did not grow well. This derivative had a DLX-MIC of 0.125 mg/L (a fourfold increase compared to the parental isolate), and there was no substantial difference in MIC-fold reduction for DLX and CIP after adding PaβN (each twofold reduction in MIC).

The effect of introducing the wild-type efflux-related genes was limited; the highest was for DLX-MIC in the derivatives 32D3, 34D1, and 37D2, with an 8-, 4-, and 5.4-fold reduction of the MICs for DLX and a twofold reduction for CIP in all derivatives (Table 2) by introducing wt-*emrR*, suggesting a role of the mutations C251A (S84Y), C331T (R111C), and A326C (D109A) as potentially causative for DLX resistance evolution. Then, 34D1 and 37D2 harbored additional mutations in *marR*, A214C (T72P) and G206A (G69E), respectively. The introduction of wt-*marR*, however, did not significantly reduce DLX-MIC in 37D2; in 34D1, a fourfold reduction of MIC was seen for both DLX and CIP. The highest reduction in CIP-MIC (eightfold) was seen by introducing the wt-*marR* into 32D2, displaying T39A, whereas DLX-MIC only dropped by twofold. The introduction of wt-*mdtC* into 34D1, harboring V832I, led to a fourfold DLX-MIC reduction without affecting the MIC of CIP. This was not reproducible in 34D3, a second clone of the same parental isolate, harboring V832I, where the introduction of wt-*mdtC* did not affect the MICs of DLX or CIP. Then, 34D3, in comparison to 34D1, lacked *marR* mutations and harbored Q138K in *emrR* instead of R111C. As the derivatives 34C1 and 34C2 harbored a similar mutation in *marR* (G69E) and *mdtC* (V632I), respectively, as detected in DLX-challenged strains at pH 6.0, these derivatives were included in the complementation to examine their effect on the MIC (Table 2). We also examined *parC* for certain derivatives, as mutations at the A117 site were the commonality between DLX- and CIP-challenged strains. The wt-gene substantially reversed resistance for both DLX and CIP, suggesting this mutation to be responsible for DLX- as well as CIP-resistance in *E. coli*. In addition, we examined *gyrA* mutations in strains for which we had observed DLX resistance. We were able to introduce *wt-gyrA* into the strains harboring C248T (S83L), A260G (D87G), G1846A (G616S), and C1841T (A614V). The only difference was seen for G616S, for which the introduction of the *wt*-gene led to a fourfold reduction of DLX-MIC but not CIP-MIC. Cloning the shuttle vector PUCP24, without candidate genes, into different derivatives did not lead to a change in DLX- or CIP-MIC.

**TABLE 2:** Effect of introduction of wild-type genes into selected *E. coli* derivatives on DLX- and CIP-MICs^*c*^

		Culturing conditions				Parental strain (PS)		Derivative strain (DS)		Complement strain (CS)		Fold change in MIC (DS compared to PS)^*a*^		Fold change in MIC (DS compared to CS)^*b*^	
Introduced wt-gene	ID of derivative	Antibiotic	pH	Nucleotide change	Amino acid change	DLX-MIC	CIP-MIC	DLX-MIC	CIP-MIC	DLX- MIC	CIP- MIC	DLX-MIC fold change	CIP-MIC fold change	DLX-MIC fold change	CIP-MIC fold change
gyrA	31D3	DLX	7.3	C1841T	A614V	0.047	0.008	2	0.25	2	0.125	42.6	31.25	1	2
gyrA	34D3	DLX	7.3	A260G, C248T	D87G, S83L	0.064	0.016	32	2	8	0.5	500	125	4	4
gyrA	36D2	DLX	7.3	C248T	S83L	0.016	0.008	2	0.5	2	0.25	125	62.5	1	2
gyrA	34D1	DLX	6	G1846A	G616S	0.008	0.032	0.25	0.25	0.064	0.25	31.25	8	4	1
gyrA	34D3	DLX	6	C248T	S83L	0.008	0.032	1	4	0.5	4	125	125	2	1
emrR	31D1	DLX	6	G329A	R110H	0.016	0.064	0.5	0.5	0.25	0.25	31.25	8	2	2
emrR	34D3	DLX	6	C412A	Q138K	0.008	0.032	1	4	0.5	4	125	125	2	1
emrR	37D2	DLX	6	A326C	D109A	0.016	0.032	0.125	0.125	0.023	0.064	8	4	5.4	2
emrR	39D2	DLX	6	C331A	R111S	0.004	0.064	0.032	0.064	0.023	0.064	8	1	1.4	1
emrR	34D1	DLX	6	C331T	R111C	0.008	0.032	0.25	0.25	0.064	0.125	31.3	8	4	2
emrR	32D3	DLX	7.3	T418A	W140R	0.064	0.008	16	4	8	2	250	500	2	2
emrR	31D3	DLX	7.3	T476C	L159P	0.047	0.008	2	0.25	1	0.25	42.6	31.25	2	1
emrR	34C2	CIP	7.3	A166G, T172A	T56A, F58I	0.064	0.016	0.5	0.5	0.25	0.5	8	31.25	2	1
emrR	34D3	DLX	7.3	T452A	L151H	0.064	0.016	32	2	16	0.5	500	125	2	4
emrR	36D2	DLX	7.3	A326G	D109G	0.016	0.008	2	0.5	2	0.25	125	62.5	1	2
emrR	32D3	DLX	6	C251A	S84Y	0.016	0.064	2	8	0.25	4	125	125	8	2
marR	31C3	CIP	6	GAA253-255ATT, G258T, T260G	E85I, R86S, L87W	0.016	0.064	2	16	1	8	125	250	2	2
marR	34C1	CIP	7.3	G206A	G69E	0.064	0.016	1	1	0.5	0.5	16	62.5	2	2
marR	31D1	DLX	6	G205A	G69R	0.016	0.064	0.5	0.5	0.25	0.25	31.25	8	2	2
marR	31D3	DLX	6	C217T	R73C	0.016	0.064	1	1	0.5	0.5	62.5	16	2	2
marR	32D2	DLX	6	C217T, AT220-221GG	R73C, M74G	0.016	0.064	0.125	0.25	0.032	0.064	8	4	4	4
marR	34D1	DLX	6	G206A	G69E	0.008	0.032	0.25	0.25	0.064	0.064	31.25	8	4	4
marR	37D2	DLX	6	A214C	T72P	0.016	0.032	0.125	0.125	0.064	0.094	8	4	2	1.3
marR	32D3	DLX	7.3	A115G	T39A	0.064	0.008	16	4	8	0.5	250	500	2	8
marR	31D3	DLX	7.3	T233C	L78P	0.047	0.008	2	0.25	1	0.125	42.6	31.25	2	2
mdtC	34D1	DLX	6	G1894A	V632I	0.008	0.032	0.25	0.25	0.064	0.25	31.25	8	4	1
mdtC	34D3	DLX	6	G1894A	V632I	0.008	0.032	1	4	0.25	4	125	125	4	1
mdtC	34D3	DLX	7.3	G1894A	V632I	0.064	0.016	32	2	24	1	500	125	1.3	2
mdtC	34C2	DLX	7.3	G1894A	V632I	0.064	0.016	0.5	0.5	0.25	0.5	8	31.25	2	1
parC	36D2	DLX	7.3	C350T	A117V	0.016	0.008	2	0.5	0.25	0.064	125	62.5	8	8
parC	32D3	DLX	6	C1690A	R564S	0.016	0.064	2	8	2	16	125	125	1	0.5
soxR	33D1	DLX	7.3	A175G, C437T	I59V, A146V	0.064	0.016	16	8	4	2	250	500	4	4

^*a*^ Calculated as MIC of the derivatives divided by the MIC of the parental isolates.

^*b*^ Calculated as MIC of the derivatives divided by the MIC of the complemented isolates. Amino acids are depicted as one letter code.

^*c*^ Wt: wild-type, DLX: delafloxacin, CIP: ciprofloxacin.

### Efflux assays

**FIG 4:**
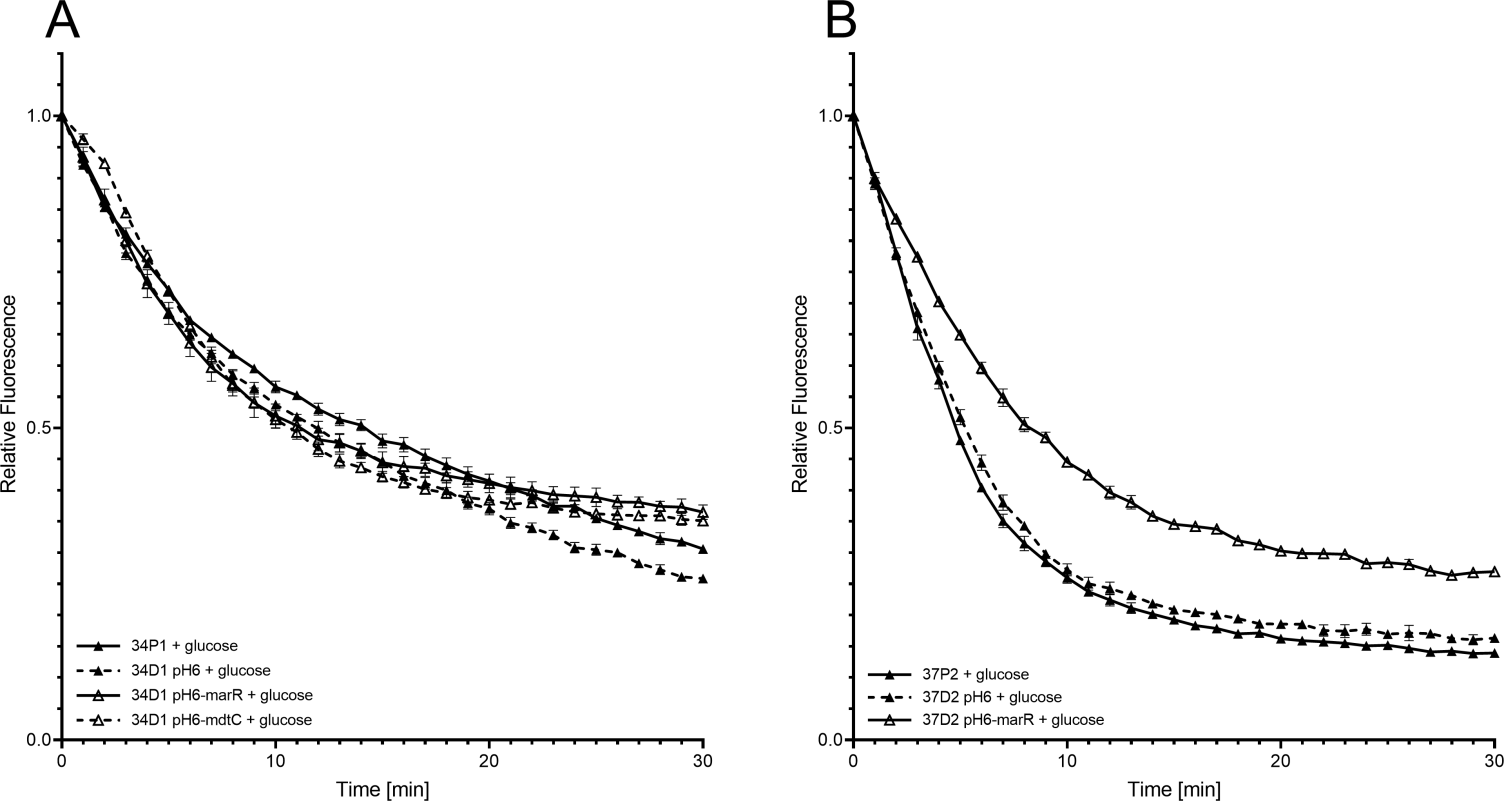
Efflux of EtBr from isolates 34 and 37. Efflux of EtBr in the presence of glucose and the absence of the putative efflux pump inhibitor chlorpromazine. 34P1 and 37P2, parental isolates; 34D1 pH6 and 37D2 pH6, resistant derivatives; 34D1 pH6-marR, 34D1 pH6-mdtC, and 37D2 pH6-marR, resistant derivatives with the introduced respective wt gene (transformants). (**A**) Isolate 34. (**B**) Isolate 37.

As the main difference in MIC reduction for DLX and CIP by introducing the wt-gene was observed for *emrR* in resistant derivatives 37D2 and 34D1, these were used for further efflux assays. For isolate 37, there was no substantial difference in efflux of ethidium bromide (EtBr) between the parental isolate and the resistant derivative (37D2) when glucose was added (Fig. 4A and B), questioning the role of D109A in *emrR* and T72P in *marR* in efflux overexpression. Efflux was slower in transformants harboring the respective introduced wt-gene, suggesting an additional transcriptional repression of the EmrABTolC pump (21) and the marRAB operon (22).

For isolate 34, we observed a slightly increased efflux in the resistant derivative (34D1). The transformant harboring the wt-*emrR* did not revert the efflux intensity, suggesting a role for V632I in *mdtC* or G69E in *marR* for efflux upregulation.

## DISCUSSION

This study sought to examine the resistance evolution of DLX and CIP in *E. coli* and cross-resistance evolution at pHs 6.0 and 7.3. We observed a substantially slower resistance development for DLX at pH 6.0 than at pH 7.3. The opposite was the case for CIP, where resistance evolved faster at pH 6.0 than at pH 7.3. Penetration of DLX into bacterial cells is enhanced in an acidic environment (2, 23). Acidic pH is present at many infection sites, such as urinary tract infections (intraabdominal), abscesses, and biofilms (6 – 12, 24), where *E. coli* might be one of the causative pathogens. The enhanced penetration into bacteria, together with the slower resistance evolution in an acidic environment, might display DLX as a valuable compound for these infections with *E. coli*.

Cross-resistance to DLX was more frequently observed for strains cultured in subinhibitory concentrations of CIP, with the majority occurring at pH 7.3, whereas cross-resistance to CIP in those challenged with DLX was less frequent. Concluding that CIP might still be susceptible in clinical *E. coli* that have evolved DLX resistance *in vivo*, whereas this will probably not be the case vice versa. For clinical applicability, however, more *in vivo* and future real-world clinical data are needed.

In all resistant derivatives examined, CIP was selected for mutations in *gyrA*, all comprising mutations at the S83 and D87 sites, which are two mutational hotspots for quinolone resistance, located on the GyrA helix-4 (18). A complete selection of *gyrA* mutations was not observed for DLX, which was in addition selected for mutations outside the conserved QRDR region (19). We identified G616S in *gyrA*, involved in DLX resistance, as its complementation led to a fourfold reduction of MIC for DLX but did not change the MIC for CIP.

The causative relationship between the mutation A117V in *parC* and resistance to CIP as well as DLX was demonstrated by the complementation with the wild-type *parC* into the resistant derivatives, which substantially reduced MIC for both CIP and DLX.

As the most significant effect of efflux pump inhibition was seen for DLX-MIC in DLX-challenged clones at pH 6.0, complementation was mainly performed for efflux-related genes of these derivatives. The main difference in efflux-related genes between DLX- and CIP-challenged derivatives were *emrR* mutations that were predominantly selected by DLX. A causative relationship for DLX resistance was observed for S84Y, D109A, and R111C, for which the introduction of the wild-type genes reduced the DLX-MIC by 8-, 5.4-, and 4-fold, respectively. EmrR is a repressor gene, regulating the expression of the EmrAB multidrug transport system in *E. coli* (21). Mutations at the D109 site in *emrR*, among others, have previously been selected in *E. coli* that were exposed to pH 6.5 and carbonyl cyanide m-chlorophenylhydrazone (CCCP), which is a strong uncoupler of oxidative phosphorylation (25). The EmrAB multidrug efflux pump exports CCCP from the cell, and the expression of EmrAB is thought to be induced by this uncoupler. qPCR revealed an overexpression of *emrA* and *B* and *tolC* in our tested derivatives. Although the gene expressions compared to the parental isolates were significant (17), the maximum fold expression was 8.6-fold (*emrA*). This modest overexpression, together with the results of the efflux assays, suggests an additional mechanism of this regulator gene in DLX resistance. Indeed, previous reports suggest that EmrAB-TolC plays no relevant role in mediating resistance to quinolones (17, 26 – 28). Rodionov et al. have identified *nmpC* as a possible member of the emrRAB operon, observing co-regulation of EmrAB and this porin gene (29). NmpC is an outer membrane porin that forms pores in lipid bilayers and contributes to the heat resistance of *E. coli* (30). Its role in antibiotic resistance, however, is unclear. Negative regulation of *nmpC* during growth at low pH has been observed by Coll et al. (31). Given the strong EmrR boxes upstream of *nmpC* (29), there is a possibility that the DLX resistance of our *emrR* mutants results from regulation of NmpC.

MarR is a repressor of the mar operon, and mutations in *marR* promote the overexpression of the AcrAB efflux pump (22), which is involved in the active efflux of quinolones (32).

The role of AcrAB-TolC in resistance to DLX has been shown for progenies of persister cells and stationary-phase *E. coli* by Byrd et al. (33), who showed that deletion of *acrB* reduced persistence in colony biofilms and loss of AcrAB-TolC function decreased resistance development to DLX. Our efflux experiments suggest a possible role of the G69E *marR* mutation in DLX resistance. However, although complementation for this mutation led to a twofold reduction of DLX-MIC, it did not result in major differences in effects on CIP-MIC, suggesting a role for this mutation for both DLX and CIP resistance.

Our efflux assays indicate a possible role for efflux-mediated resistance via V632I in *mdtC*, which encodes the MdtC subunits in the heterotrimeric efflux transporter MdtB_2_C complex of *E. coli*, belonging to the RND family (34) and can confer low-level norfloxacin resistance (35). The effect of complementation of V632I on DLX-MIC was only seen in the derivatives grown at pH 6.0 but was not reproducible in their clones grown at pH 7.3, suggesting a role of pH in the expression of the *mdtC* gene.

One of the reasons that the MICs in our derivatives did not fully reverse when complementing efflux-related alleles might be due to the fact that the levels of quinolone resistance rising due to regulatory mutation and pump overexpression are often limited to four- to eightfold increases in MICs, likely because of counterbalancing regulatory factors and cellular toxicities of high levels of pump overexpression (36).

One of the limitations of this study is that the majority of the *E. coli* isolates chosen were sampled from urinary tract infections. Therefore, we cannot generalize our findings to other infection niches where acidic pH is predominant as well, such as abdominal abscesses or biofilms. Further experiments are needed to unravel the resistance mechanisms of *E. coli* to DLX and to confirm the role of *nmpC* for DLX resistance.

### Conclusion

This study identifies a multifactorial mechanism for the resistance evolution of delafloxacin in *E. coli*, which involves new mutational sites in topoisomerase genes and mutations in efflux-related genes. Compared to ciprofloxacin, resistance to delafloxacin evolved much slower in slightly acidic than in neutral pH. This highlights the importance of understanding micro-environmental conditions at infection sites that might affect the true clinical efficacy of antibiotics and challenges our current antibiotic susceptibility-testing paradigm. In addition, delafloxacin might be a valuable candidate for the treatment of Gram-negative pathogens in abscesses and biofilms, due to its potency in acidic pH. More *in vitro* as well as *in vivo* experiments are needed to define the role of delafloxacin for Gram-negative pathogens and to identify the best infection-target sites for this novel antibiotic.

## MATERIALS AND METHODS

### Bacterial isolates and culture conditions

Eight clinical *E. coli* isolates, collected from two different clinical sites (urine: *n* = 7, blood culture: *n* = 1), were used for multistep resistance selection.

Bacterial cells were cultured in 5 mL Mueller Hinton 2 Broth (MHB) (Sigma-Aldrich Chemie GmbH, Schnelldorf, Germany) on Nunc CELLSTAR 6-well plates (Greiner Bio-One GmbH, Frickenhausen, Germany) with shaking at 120 rpm on a microplate shaker (Edmund Bühler GmbH, Germany) at 37°C.

DLX and CIP were purchased from Sigma-Aldrich Chemie GmbH, and stock solutions were made by dissolving powder stocks according to the manufacturer’s instructions in 0.2 µm filter-sterilized water and stored at −20°C before use. MHB was used at a standard pH of 7.3 and adjusted to an acidic pH of 6.0 by the dropwise addition of 1 M hydrochloric acid-HCl (Sigma-Aldrich Chemie GmbH). pH measurement was performed with SevenEasy pH (Mettler-Toledo GmbH, Schwarzenbach, Switzerland).

### MIC determination/antimicrobial susceptibility testing

MICs for several antibiotics were determined by broth microdilution in MHB using the Sensititre GNX2F plates (Thermo Fisher Scientific, East Grinstead, United Kingdom). Isolates that were incubated in acidic conditions were also tested under acidic conditions with MHB adjusted to pH 6.0. MICs for DLX and CIP were additionally determined by the broth microdilution method (range of each antibiotic 0.002–128 mg/L) on 96-well plates (Nunc CELLSTAR Greiner Bio-One GmbH, Frickenhausen, Germany) with a final volume of 100 µL per well and an inoculum of 5 × 10^5^ colony-forming unit (CFU)/mL (37) with the pH of the broth adjusted to the pH that was used during the passages.

MIC determination was done following the latest EUCAST recommendations (15), and values were interpreted in accordance with the 2022 EUCAST criteria (version 12.0, 2022).

### MBC determination

Broth dilution MBCs were determined by first performing the standard broth microdilution technique for MIC as described above and by using a 99.9% threshold for bacterial killing, as recommended by the CLSI (38). Growth was examined by plating broth on nonselective agar plates. Briefly,when determining the MBCs, 10 µL from the well with the lowest concentration of antibiotic without visible growth as well as the next two higher concentrations was transferred to nonselective Luria-Bertani agar (LBA) plates and incubated at 37°C (38). After 24 and 48 h, the plates were inspected, and visible colonies were counted. Since the initial concentration was 5 × 10^5^ CFU/mL, the lowest concentration showing <5 CFU/plate bacterial growth on LBA was reported as MBC (39). Consequently, the MBC was deemed the minimum antimicrobial concentration capable of inactivating more than 99.9% of the bacteria present.

In order to ensure that DLX and CIP were diluted to subinhibitory doses before the bacteria were transferred to and allowed to grow on LBA, we repeated MBC measurements as described above, with an additional washing step. Briefly, before transferring broth to nonselective agar plates, the broths were centrifuged at 6,000 rpm for 5 min, and a washing step with phosphate buffered solution (PBS) was performed before 10 µL of the broth was plated on the nonselective agar for incubation.

### Efflux pump inhibition assay

MICs were also determined in the presence of the efflux pump inhibitor PaβN (Sigma-Aldrich, Steinheim, Germany). Here, broth microdilutions of the resistant derivatives were performed on 96-well plates (Nunc CELLSTAR Greiner Bio-One GmbH, Frickenhausen, Germany) subjected to 25 mg/L of PaβN (40) plus 1 mM MgSO_4_ to stabilize the outer membrane in case of the permeabilizing effect of PaβN (41). The efflux pump inhibition assay was also performed with MHB at pH 7.3 or 6.0, depending on the pH the isolates were passaged at. In order to measure the differences in how PAβN affects the MIC in both parental strains and their resistant derivatives, the ratio of the MIC reduction was calculated by dividing the MIC reduction in the derivatives by the MIC reduction in parental isolates after adding PAβN.

### Statistical analysis

To determine the influence of the pH on resistance development, the fraction of isolates exhibiting an MIC above the respective breakpoint was compared between isolates tested at neutral and acidic pHs using χ. To determine the influence of the efflux pump inhibitor, the means of all MIC/MIC 25 mg/L PaβN ratios per isolate were compared between neutral and acidic pHs using an unpaired *t*-test; two-tailed *P*-values of ≤0.05 were considered statistically significant. GraphPad Prism v 8.3.4 was used for statistical analyses.

### Experimental evolution for antimicrobial resistance (multi-step resistance selection)

Three clones of each of the eight parental *E. coli* isolates were selected by picking 1 CFU and re-streaking on a new Mueller Hinton (MH) agar plate (Fig. 1C). From this plate, three colonies were chosen that served as parental clones for the passaging experiments. MIC measurements for DLX and CIP were separately performed for each clone, measured either at pH 6.0 or 7.3 (depending on the pH they would be subjected to). The clones were subjected in parallel to subinhibitory concentrations (i.e., 1/2 MIC of each strain) of DLX or CIP in either neutral MHB (pH 7.3) or acidic MHB (pH 6.0). Briefly, one single colony of an *E. coli* clone grown overnight at 37°C on an MH agar plate was picked and grown in 5 mL of MH broth at 37°C for 18 h without antibiotics (overnight). Twenty-five microliters of the overnight broth was transferred to new MHB (with respective pH at either 6.0 or 7.3) containing CIP or DLX at 1/2 of the initial MICs of the strain and incubated at 37°C with shaking at 120 rpm. Twice a day, 25 µL of the previous bacterial culture was transferred to fresh MH broth with adjusted pH and antibiotics (first passage) in order to correct for the change of pH during incubation , which showed an approximation of the pH values after 6 h of incubation. After 3 days of being subjected to the same antibiotic concentration, aliquots were sampled, washed with PBS to remove antibiotics, and re-suspended in MHB, and 25 µL was kept in 1 mL of 10% glycerol LB broth at −80°C for further molecular investigations and the next round of passages. MICs for DLX and CIP were re-tested by broth microdilution, and the antibiotic concentration for the next passages was adjusted accordingly. A total of 96 clones were subjected to either DLX or CIP at pH 7.3 or 6.0, respectively (24 clones were subjected to DLX at pH 6.0, 24 to DLX at pH 7.3, 24 to CIP at pH 7.3, and 24 to CIP at pH 6.0). The same procedure was also performed in parallel without antibiotic pressure (negative controls).

### Genomic DNA isolation

Total DNA was extracted from the strains with the QiaAMP Mini Kit (QIAGEN, Hilden, Germany) following the manufacturer’s instructions. DNA concentrations were measured with a Nanodrop OneC spectrometer (Thermo Fisher Scientific, Massachusetts, USA).

### Illumina library preparation

One nanogram of DNA from each sample was tagmented using Illumina Nextera XT according to standard protocol. Nextera adapters containing Unique Dual Indices were added by PCR. The libraries were double-sided size selected, and the quality and quantity of the libraries were validated using the Fragment Analyser (Agilent, Santa Clara, CA, USA). The libraries were normalized to 10 nM in Tris-Cl 10 mM, pH 8.5, with 0.1% Tween 20, and pooled equimolar.

### Cluster generation and illumina sequencing

After library quantification, libraries were prepared for loading according to the NovaSeq workflow with the NovaSeq 6000 Reagent Kit (Illumina, Catalog No. 20012865).

Cluster generation and sequencing were performed on a NovaSeq 6000 System with a run configuration of paired end (PE) at 2× 150 bp.

### Variant analysis

Illumina PE reads were quality checked using FastQC (v0.11.9) (42) and FastQScreen (v0.14.1) (43). Adapter sequences and low-quality read ends (identified with a sliding window of 4 bp, where the base quality is lower than Q20) were trimmed away using Fastp (v0.20.0) (44). Trimmed reads were quality (Q20) and length (18 bp) filtered using the same tool. Trimmed and filtered reads were mapped to the reference genome (Ensembl *Escherichia_coli* K12 MG1655 ASM584v2) using bowtie2 (v2.4.2) (45).

For each experiment, variants in related samples were identified using samtools (v1.11) and bcftools (v1.11) (46) and functionally annotated using SnpEff (v4.3) (47). SNPs with variant quality above Q20 were extracted using vcftools (v0.1.16) (48). High-quality SNPs specific to each evolved clone were identified using SnpSift in SnpEff (v4.3) (47) by comparison against the parental clone.

### Complementation assays

To evaluate the impact of the mutations on DLX and CIP susceptibility, a series of cloning and expression experiments were performed.

The candidate genes from parental isolates and their putative Shine-Dalgarno sequence were PCR amplified with primers designed with the Takara In-Fusion Cloning Primer Design Tool to contain the desired restriction sites (Table 3). The amplicons and the shuttle vector pUCP24 plasmid were digested with the corresponding restriction enzymes, purified, ligated with Quickligase (New England Biolabs, Ipswich, USA), and then transformed into competent *E. coli* (Top10 Mix & Go; Zymo Research, USA). For cloning *gyrA* into PUCP24, we used In Fusion Snap (Takara Bio, California, USA) in order not to digest the amplicon because the restriction sites we used were present in *gyrA*. White transformants were selected on 30 mg/L gentamycin LB agar plates treated with X-gal and isopropyl-β-D-thiogalactopyranosid, and the insertion of the gene was confirmed by sequencing. The recombinant plasmids were extracted with the NucleoSpin Plasmid Kit (Macherey-Nagel, Düren, Germany), and the insertion was confirmed by sanger sequencing (Microsynth, AG, Balgach, Switzerland). Afterward, they were transformed into the selected derivatives that were made competent with the Mix & Go *E. coli* Transformation Kit & Buffer Set (Zymo Research) according to the provider’s instructions. Transformants were selected on 30 mg/L gentamycin LB agar plates. The DLX and CIP resistance of transformants was determined by broth microdilution. As a negative control, the shuttle vector pUCP24 without candidate genes was introduced to the strains to analyse the effect of the vector on the MICs of the antibiotics.

**TABLE 3:** Primers used in this study^*a*^

Primer Name	Primer Sequence (5’ – 3’)	Function	Source
marR-F1 marR-R1	GCAGGTCGAC**TCTAGA**CGTCATACCTCTTTTTTGTTTACGG ATGATTACGAATTC**GAGCTC**CGATTTAGCAAAACGTGGCATCG	Amplification of marR	This study
emrR-F1 emrR-R1	ATGATTACGAATTC**GAGCTC**CCCACAAGAATCATTTTTCTAAAAC GCAGGTCGAC**TCTAGA**TAAATCTGGATTTTTGAGCGAGATG	Amplification of emrR	This study
mdtC-F1 mdtC-R1	ATGATTACGAATTC**GAGCTC**GGTGCTGACGCTGTTTACCACG GCAGGTCGAC**TCTAGA**CCAACGGGTGCTGTCGGG	Amplification of mdtC	This study
parC-F1 parC-R1	GAATTC**GAGCTC**TTCACGTTGATCTCCTGTGACTCG GTCGAC**TCTAGA**GCGGGAGGAAACAGCGCC	Amplification of parC	This study
gyrA-F1 gyrA-R1	GCAGGTCGAC**TCTAGA**ATAGCTCCCTTTTGGCATGAAG ATGATTACGAATTC**GAGCTC**ATTGGATGTGAATAAAGCGTATAGG	Amplification of gyrA	This study
emrA-F1 emrA-R1	GCAGCAACCGGTAAAGAAGA TCGAAGTGACGCAGTACCAA	RT-PCR	(17)
emrB-F1 emrB-R1	GACCGACGATAACCCGATAGT AATAGCGCCGAAGTAGAGCA	RT-PCR	(17)
tolC-F1 tolC-R1	GCAAGCACGCCTTAGTAACC CACTGGTCGCGTTAGAGTTG	RT-PCR	(17)
cysG-F1 cysG-R1	TTGTCGGCGGTGGTGATGTC ATGCGGTGAACTGTGGAATAAACG	RT-PCR	(49)

^*a*^ Restriction sites SacI (GAGCTC) and XbaI (TCTAGA) are highlighted in bold characters. RT-PCR, reverse transcription PCR.

### Efflux assay

Efflux measurements were performed according to Viveiros et al. (50). Briefly, strains were grown on LB agar or LB agar supplemented with 30 mg/L gentamicin (latter for transformants) overnight, transferred to LB broth, and incubated overnight. On the next morning, they were diluted to an optical density (OD_600_) of 0.1 nm and incubated until they reached midlog phase, corresponding to an OD_600_ of 0.6–0.8 on a shaker incubator. Cells were then centrifuged at 16,000 × *g* for 3 min, and pellets were washed twice with 1× PBS. The OD_600_ of the suspension was adjusted to 0.6 in 1× PBS.

Then, 1 mg/L EtBr and 20 mg/L chlorpromazine (CPZ) were added, and the isolates were incubated for 60 min at room temperature to load the strains with EtBr. After 60 min, the tubes were centrifuged, and the discarded medium was washed twice with 1× PBS. The OD_600_ of the suspension was adjusted to 0.6 in 1× PBS. Of these, 50 µL was mixed with 50 µL of either (i) 1× PBS lacking glucose; (ii) 1× PBS lacking glucose and with CPZ; (iii) 1× PBS containing a final concentration of glucose; and (iv) 1× PBS containing glucose and with CPZ to reach an end volume of 100 µL with a final concentration of 20 mg/L CPZ and 0.4% glucose in a black 96-well plate (Greiner Bio-One GmbH, Frickenhausen, Germany). Fluorescence was measured on an Infinite 200Pro M Plex Microplate Reader (Tecan Life Sciences, Grödig, Austria) for 30 min at 37°C with measurements every minute (wavelength excitation at 539 nm and emission at 600 nm). Relative fluorescence was presented as a comparison between the fluorescence observed for the strains in the presence or absence of glucose and the control in which the strains are exposed to conditions of minimum efflux (i.e., absence of glucose and presence of CPZ). Each experiment was conducted in triplicate using qPCR.

### Determination of *emrA*, *emrB*, and *tolC* gene expression

As *emrR* regulates the expression of the EmrAB multidrug transport system of *E. coli*, we performed qPCR to examine the expression of the target genes *emrA*, *emrB*, and *tolC* according to reference (17). In addition, efflux assays with EtBr might underestimate efflux level because efflux is slow and there must be a dissociation step and probably more than one efflux event (51). Briefly, midlogarithmic phase cultures (0.5 mL) were treated with the RNAprotect reagent (Qiagen, Hilden, Germany). RNA was then extracted with an RNeasy Mini Kit (Qiagen, Hilden, Germany), and the eluate was treated with DNase I (Qiagen, Hilden, Germany), used according to the manufacturer’s instructions. Reverse transcription PCR was subsequently performed using the Power SYBRGreen RNA-to-CT 1-Step Kit (Thermo Fisher Scientific, Vilnius, Lithuania) and a QuantStudio 5 Real-Time PCR System (Applied Biosystems by Thermo Fisher Scientific) at an annealing temperature of 60°C. Transcript measurements were carried out in triplicate, and measurements were repeated twice. Quantification of relative target gene expression was performed by the 2^−ΔΔCT^ method, using *cysG* as a reference, as described by reference (49). The parental and susceptible isolates were used as the calibrators.

## SUPPLEMENTAL MATERIAL

Figure S1 aac.01625-22-s0001.tif This figure depicts the percentage of the clones over time exhibiting the EUCAST breakpoint for resistance (DLX: 0.125 mg/L, CIP: 0.5 mg/L). The x-axis represents the days incubated. Y-axis: percentage (%) of isolates exceeding the EUCAST breakpoint.

Figure S2 aac.01625-22-s0002.tif MIC evolution of single *E. coli* clones from eight parental isolates. DLX, delafloxacin; CIP, ciprofloxacin. Mean with standard error of the mean (SEM) of the MIC over time of isolates incubated in subinhibitory concentrations (1/2 MIC) of delafloxacin (A, C, E, G, I, K, M, O) and ciprofloxacin (B, D, F, H, J, L, N, P) from isolates 31 (A, B), 32 (C, D), 33 (E, F), 34 (G, H), 36 (I, J), 37 (K, L), 39 (M, N), and 42 (O, P).

Figure S3 aac.01625-22-s0003.tif MIC evolution of untreated *E. coli* isolates. DLX, delafloxacin; CIP, ciprofloxacin. Mean with standard error of the mean (SEM) of the MICs for DLX and CIP over time of untreated isolates at respective pHs.

Figure S4 aac.01625-22-s0004.tif Change in MIC by adding pAbN.

Figure S5 aac.01625-22-s0005.tif dN/dS ratio of genes.

Figure S6 aac.01625-22-s0006.tif Total number of missense SNPs over time.

Figure S7 aac.01625-22-s0007.tif Change in pH over time.

Table S1 aac.01625-22-s0008.pdf Broth microdilution of derivatives.

Table S2 aac.01625-22-s0009.pdf MIC fold-reduction of parental isolates and derivatives after the addition of PAbN.

Table S3 aac.01625-22-s0010.pdf Overview of all mutations in derivatives and the effect of PaBN.

ASM does not own the copyrights to Supplemental Material that may be linked to, or accessed through, an article. The authors have granted ASM a non-exclusive, world-wide license to publish the Supplemental Material files. Please contact the corresponding author directly for reuse.
