# Supplementary figures and images for: Resistance development in Escherichia coli to delafloxacin at pHs 6.0 and 7.3 compared to ciprofloxacin
Source: Antimicrob Agents Chemother. 2023 Oct 26;67(11):e01625-22. doi: 10.1128/aac.01625-22 (PMC10649057; doi:10.1128/aac.01625-22)

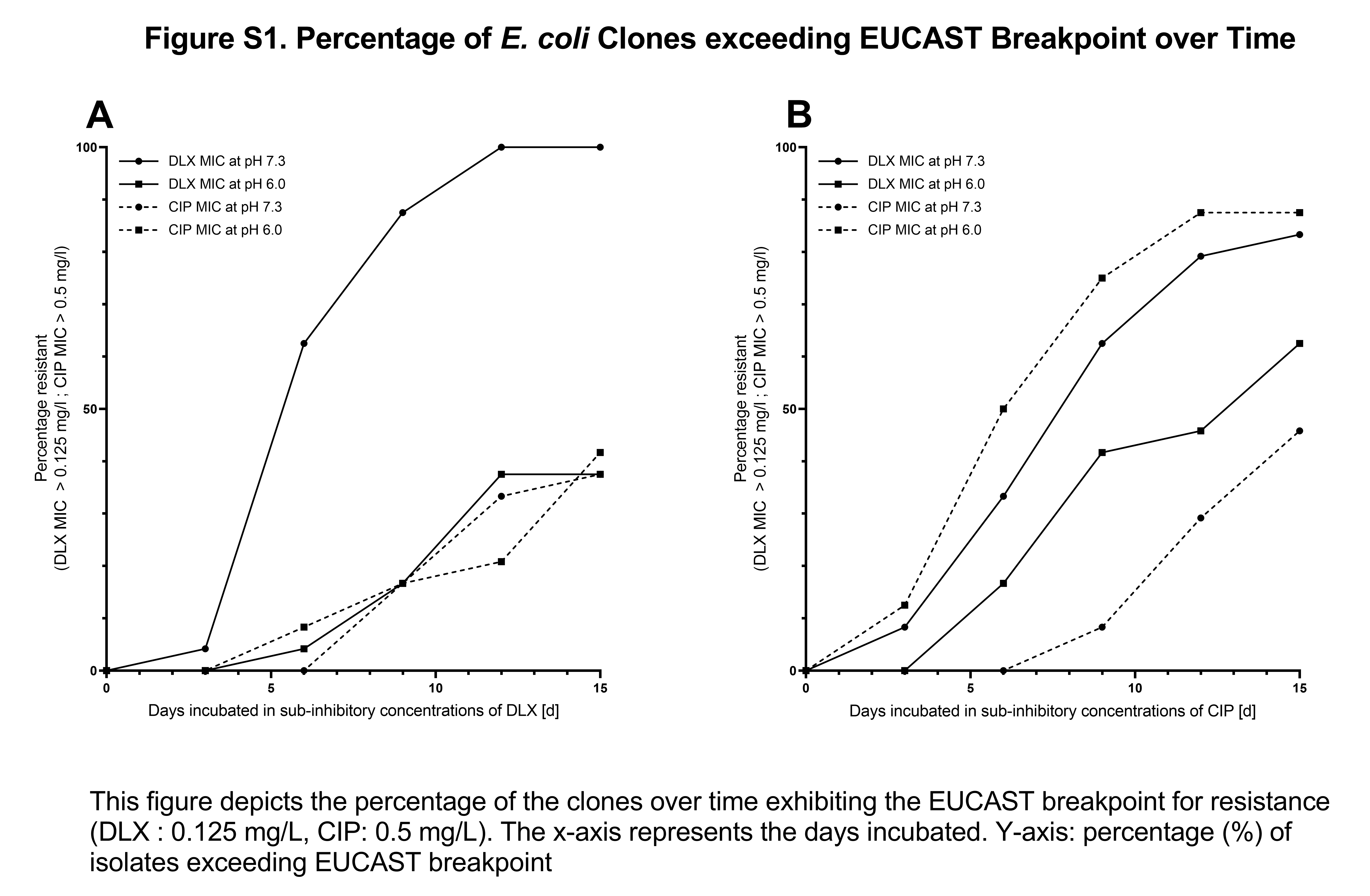

Supplement: Figure S1 — This figure depicts the percentage of the clones over time exhibiting the EUCAST breakpoint for resistance (DLX: 0.125 mg/L, CIP: 0.5 mg/L). The x-axis represents the days incubated. Y-axis: percentage (%) of isolates exceeding the EUCAST breakpoint. [file aac.01625-22-s0001.tif]

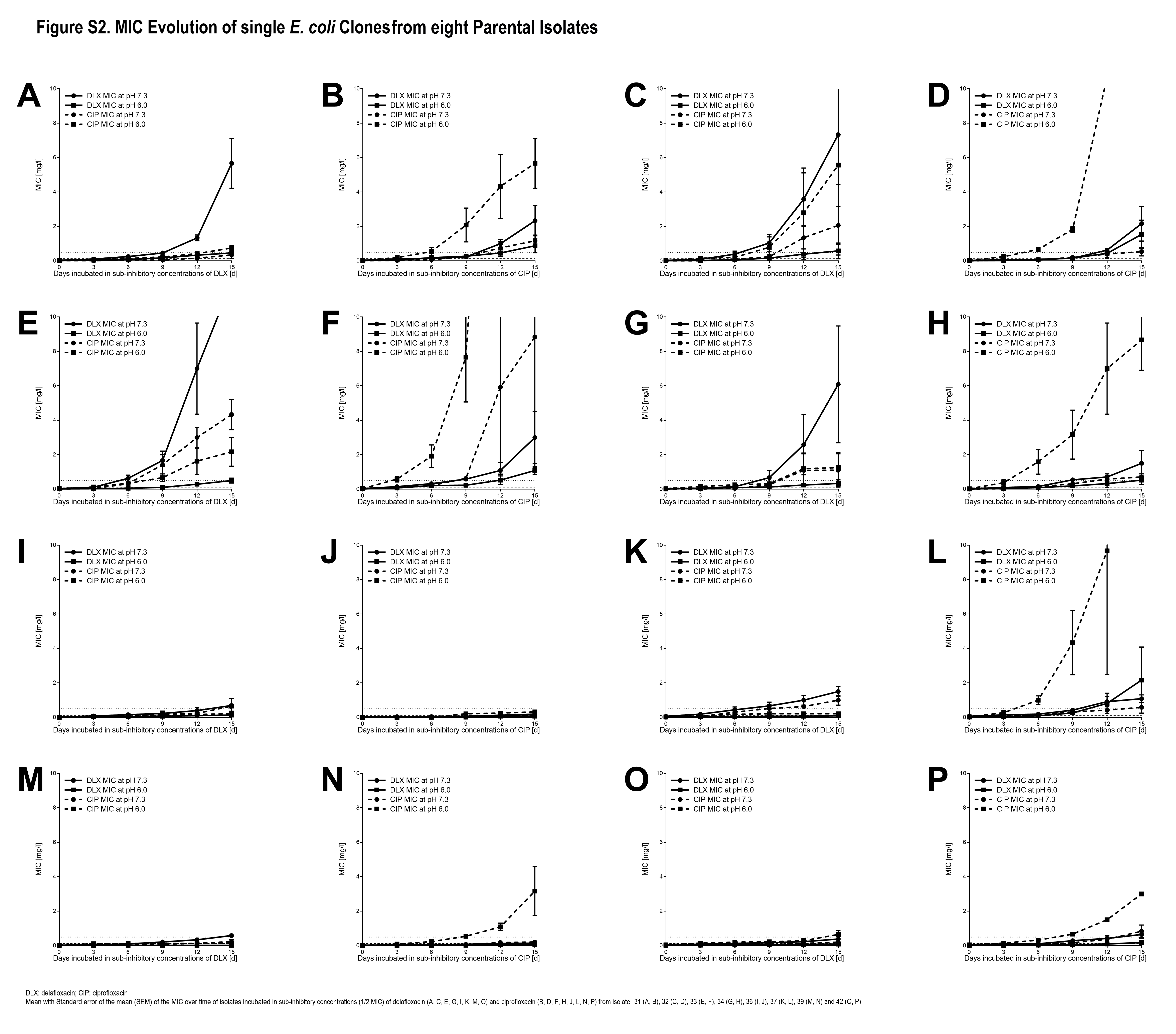

Supplement: Figure S2 — MIC evolution of single E. coli clones from eight parental isolates. DLX, delafloxacin; CIP, ciprofloxacin. Mean with standard error of the mean (SEM) of the MIC over time of isolates incubated in subinhibitory concentrations (1/2 MIC) of delafloxacin (A, C, E, G, I, K, M, O) and ciprofloxacin (B, D, F, H, J, L, N, P) from isolates 31 (A, B), 32 (C, D), 33 (E, F), 34 (G, H), 36 (I, J), 37 (K, L), 39 (M, N), and 42 (O, P). [file aac.01625-22-s0002.tif]

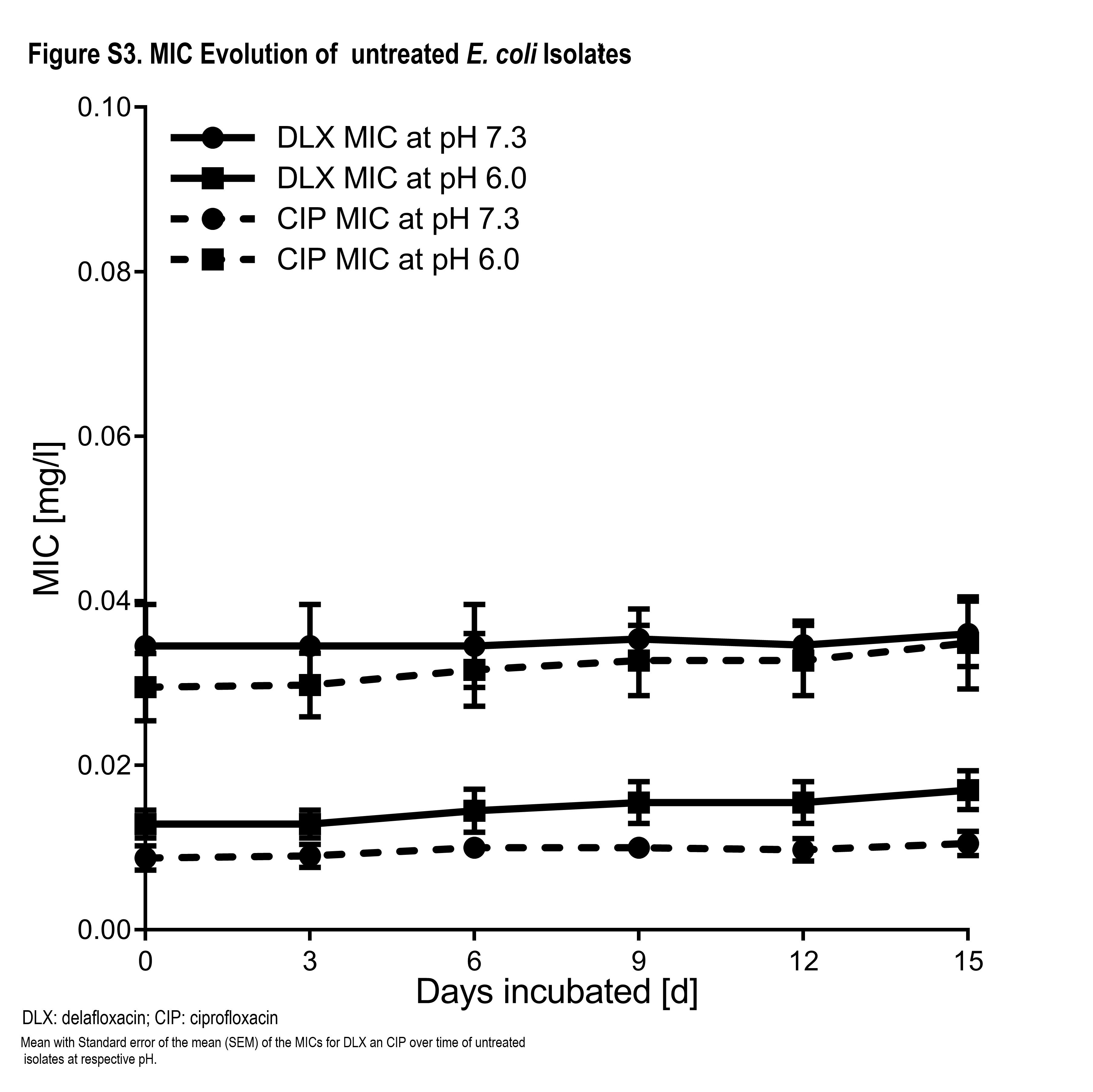

Supplement: Figure S3 — MIC evolution of untreated E. coli isolates. DLX, delafloxacin; CIP, ciprofloxacin. Mean with standard error of the mean (SEM) of the MICs for DLX and CIP over time of untreated isolates at respective pHs. [file aac.01625-22-s0003.tif]

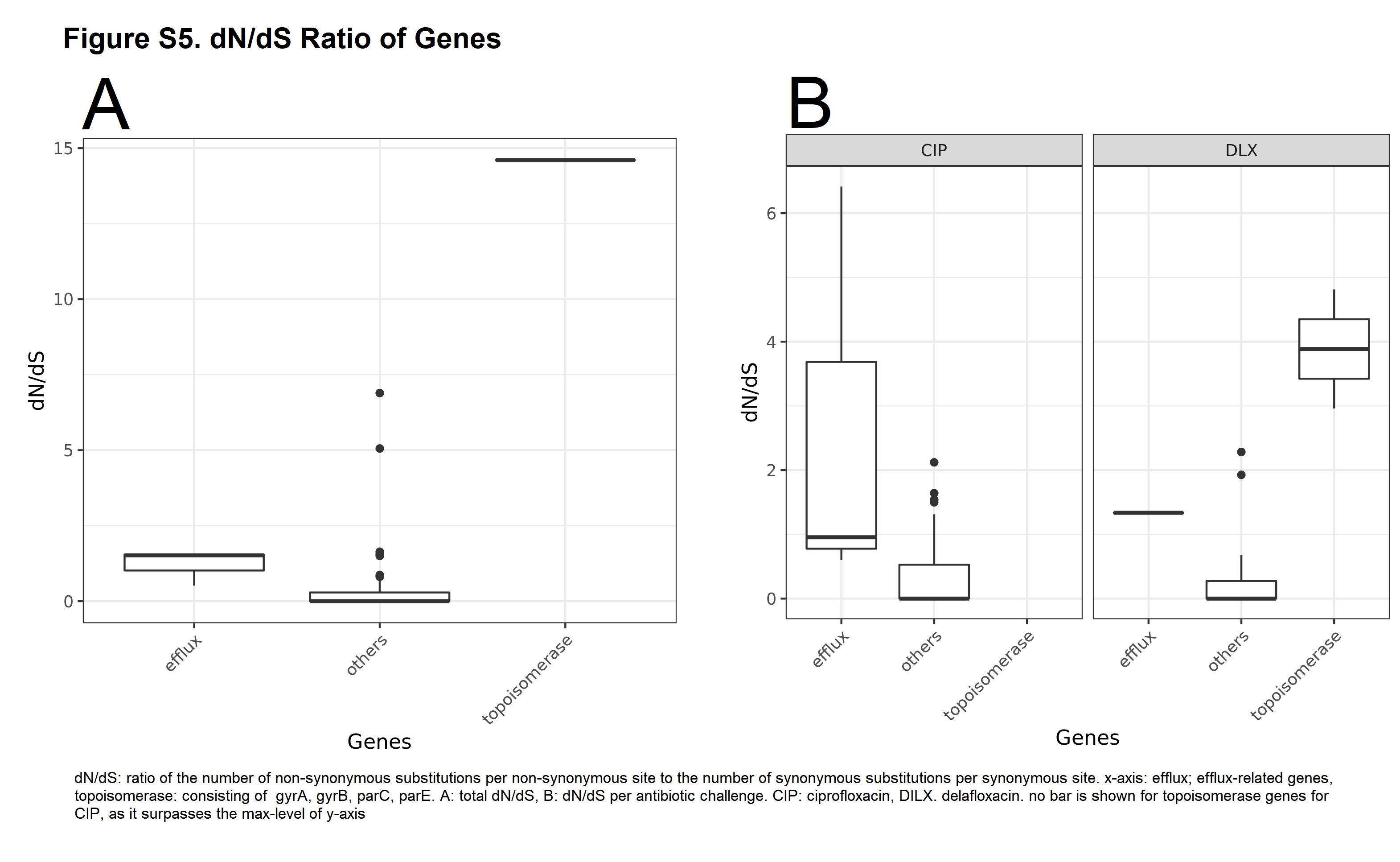

Supplement: Figure S4 — Change in MIC by adding pAbN. [file aac.01625-22-s0004.tif]

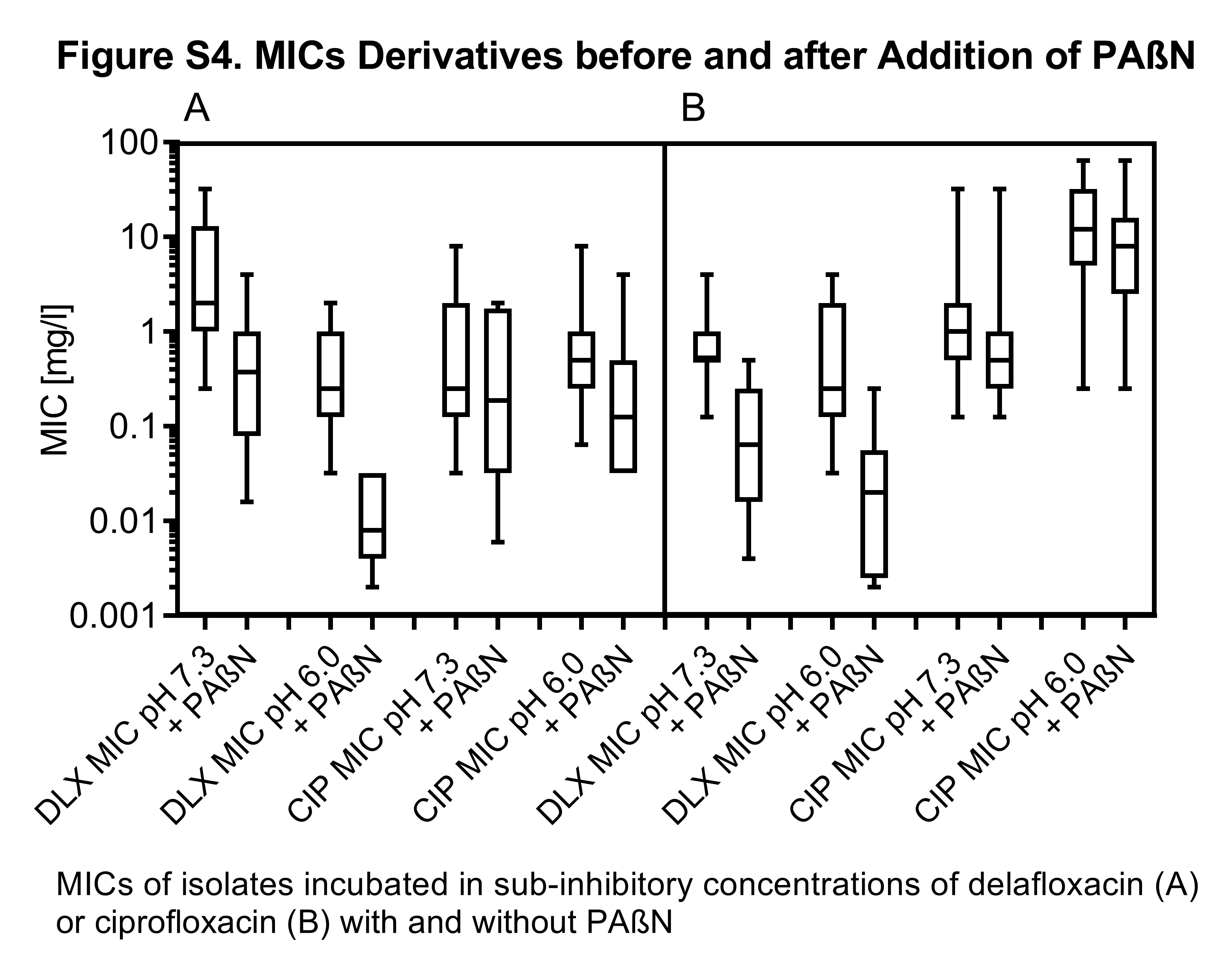

Supplement: Figure S5 — dN/dS ratio of genes. [file aac.01625-22-s0005.tif]

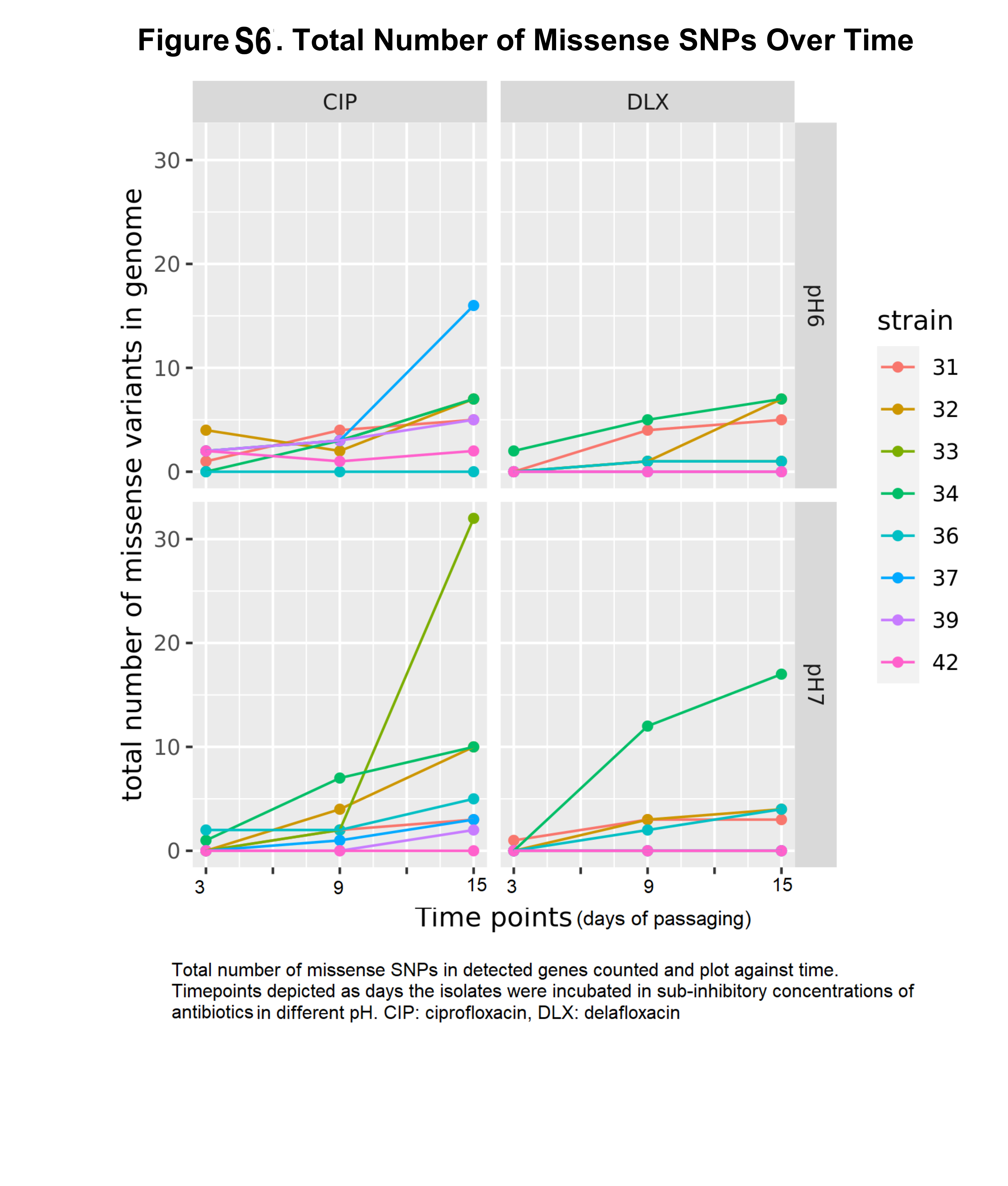

Supplement: Figure S6 — Total number of missense SNPs over time. [file aac.01625-22-s0006.tif]

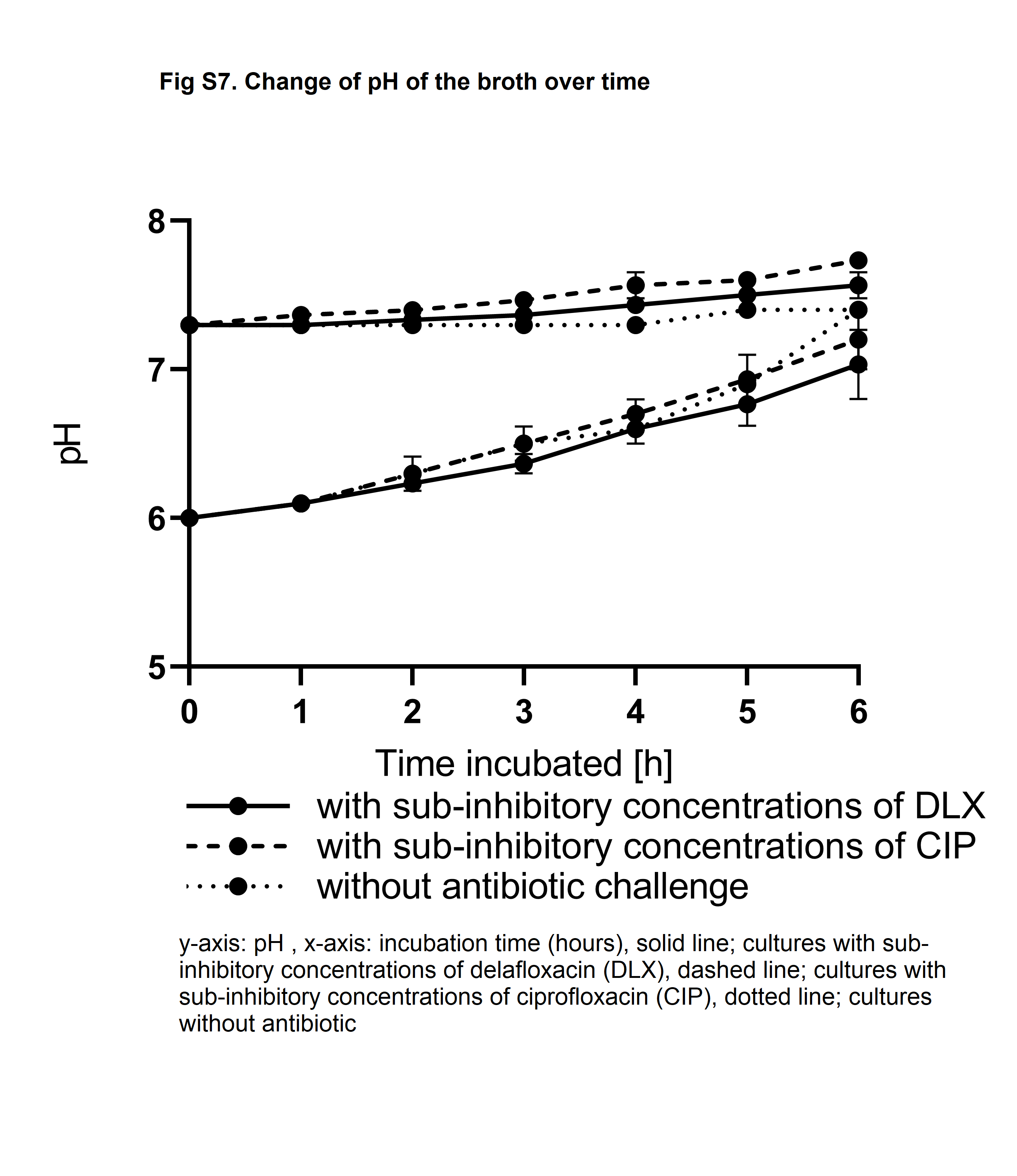

Supplement: Figure S7 — Change in pH over time. [file aac.01625-22-s0007.tif]
