# Supplementary material for: Resistance development in Escherichia coli to delafloxacin at pHs 6.0 and 7.3 compared to ciprofloxacin
Source: Antimicrob Agents Chemother. 2023 Oct 26;67(11):e01625-22. doi: 10.1128/aac.01625-22 (PMC10649057; doi:10.1128/aac.01625-22)
Supplement: Table S1 — Broth microdilution of derivatives. [file aac.01625-22-s0008.pdf]

TableS1. Broth Microdilution of Parental Isolates and respective Derivatives

		AMI	GEN	TOB	DOR	ETP	IMI	MERO	FEP	FOT	TAZ	CIP	LEVO	COL	SXT	AZT	P/T4	TIM2	DOX	MIN	TGC	POL
	S ≤	8	2	2	1	0.5	2	2	1	1	1	0.25	0.5	2	2	1	8	8	"-"	"-"	0.5	
	R >	8	2	2	2	0.5	4	8	4	2	4	0.5	1	2	4	4	8	16	"-"	"-"	0.5	
pH 7.3	31P1	≤ 4	≤ 1	≤ 1	≤ 0.12	≤ 0.25	≤ 1	≤ 1	≤ 2	≤ 1	≤ 1	≤ 0.25	≤ 1	≤ 0.25	≤ 0.5	≤ 2	≤ 8	≤ 16	≤ 2	≤ 2	≤ 0.25	≤ 0.25
	31D1	≤ 4	≤ 1	≤ 1	≤ 0.12	≤ 0.25	≤ 1	≤ 1	≤ 2	≤ 1	≤ 1	≤ 0.25	≤ 1	≤ 0.25	≤ 0.5	≤ 2	≤ 8	≤ 16	8	4	0.5	≤ 0.25
	31D3	≤ 4	≤ 1	≤ 1	≤ 0.12	≤ 0.25	≤ 1	≤ 1	≤ 2	≤ 1	≤ 1	≤ 0.25	≤ 1	≤ 0.25	≤ 0.5	≤ 2	≤ 8	≤ 16	8	8	0.5	≤ 0.25
	31C3	≤ 4	≤ 1	≤ 1	≤ 0.12	≤ 0.25	≤ 1	≤ 1	≤ 2	≤ 1	4	2	≤ 1	≤ 0.25	≤ 0.5	≤ 2	≤ 8	≤ 16	16	8	1	≤ 0.25
	32P2	≤ 4	≤ 1	≤ 1	≤ 0.12	≤ 0.25	≤ 1	≤ 1	≤ 2	≤ 1	≤ 1	≤ 0.25	≤ 1	≤ 0.25	≤ 0.5	≤ 2	≤ 8	≤ 16	≤ 2	≤ 2	≤ 0.25	≤ 0.25
	32D2	≤ 4	≤ 1	≤ 1	≤ 0.12	≤ 0.25	≤ 1	≤ 1	≤ 2	≤ 1	≤ 1	≤ 0.25	≤ 1	≤ 0.25	≤ 0.5	≤ 2	≤ 8	≤ 16	4	≤ 2	0.5	≤ 0.25
	32D3	≤ 4	≤ 1	≤ 1	≤ 0.12	≤ 0.25	≤ 1	≤ 1	≤ 2	≤ 1	≤ 1	> 2	8	≤ 0.25	≤ 0.5	≤ 2	16	≤ 16	8	4	0.5	≤ 0.25
	32C1	≤ 4	≤ 1	≤ 1	≤ 0.12	≤ 0.25	≤ 1	≤ 1	≤ 2	≤ 1	≤ 1	1	2	≤ 0.25	≤ 0.5	≤ 2	≤ 8	≤ 16	16	16	0.5	≤ 0.25
	32C2	≤ 4	≤ 1	≤ 1	≤ 0.12	≤ 0.25	≤ 1	≤ 1	≤ 2	≤ 1	≤ 1	0.5	≤ 1	≤ 0.25	≤ 0.5	≤ 2	≤ 8	≤ 16	4	≤ 2	0.5	≤ 0.25
	33P3	≤ 4	≤ 1	≤ 1	≤ 0.12	≤ 0.25	≤ 1	≤ 1	≤ 2	≤ 1	≤ 1	≤ 0.25	≤ 1	≤ 0.25	> 4	≤ 2	≤ 8	≤ 16	≤ 2	≤ 2	≤ 0.25	≤ 0.25
	33D1	≤ 4	≤ 1	≤ 1	≤ 0.12	≤ 0.25	≤ 1	≤ 1	≤ 2	≤ 1	≤ 1	> 2	8	≤ 0.25	> 4	≤ 2	≤ 8	≤ 16	8	4	0.5	≤ 0.25
	33C1	≤ 4	≤ 1	≤ 1	≤ 0.12	≤ 0.25	≤ 1	≤ 1	≤ 2	≤ 1	≤ 1	> 2	> 8	≤ 0.25	> 4	≤ 2	≤ 8	≤ 16	≤ 2	≤ 2	≤ 0.25	≤ 0.25
	33C3	≤ 4	≤ 1	≤ 1	≤ 0.12	≤ 0.25	≤ 1	≤ 1	≤ 2	≤ 1	≤ 1	1	2	≤ 0.25	> 4	≤ 2	≤ 8	≤ 16	16	8	0.5	≤ 0.25
	34P2	≤ 4	≤ 1	≤ 1	≤ 0.12	≤ 0.25	≤ 1	≤ 1	≤ 2	≤ 1	≤ 1	≤ 0.25	≤ 1	≤ 0.25	≤ 0.5	≤ 2	≤ 8	≤ 16	≤ 2	≤ 2	≤ 0.25	≤ 0.25
	34D2	≤ 4	≤ 1	≤ 1	≤ 0.12	≤ 0.25	≤ 1	≤ 1	≤ 2	≤ 1	≤ 1	≤ 0.25	≤ 1	≤ 0.25	≤ 0.5	≤ 2	≤ 8	≤ 16	≤ 2	≤ 2	0.5	≤ 0.25
	34D3	≤ 4	2	≤ 1	≤ 0.12	≤ 0.25	4	≤ 1	≤ 2	≤ 1	2	> 2	≤ 1	≤ 0.25	1	≤ 2	16	> 128	8	4	1	≤ 0.25
	34C1	≤ 4	≤ 1	≤ 1	≤ 0.12	≤ 0.25	≤ 1	≤ 1	≤ 2	≤ 1	≤ 1	1	≤ 1	≤ 0.25	1	≤ 2	8	128	8	8	0.5	≤ 0.25
	34C2	≤ 4	≤ 1	≤ 1	≤ 0.12	≤ 0.25	≤ 1	≤ 1	≤ 2	≤ 1	≤ 1	≤ 0.25	≤ 1	≤ 0.25	≤ 0.5	≤ 2	≤ 8	128	≤ 2	≤ 2	≤ 0.25	0.5
	36P2	≤ 4	≤ 1	≤ 1	≤ 0.12	≤ 0.25	≤ 1	≤ 1	≤ 2	≤ 1	≤ 1	≤ 0.25	≤ 1	≤ 0.25	≤ 0.5	≤ 2	≤ 8	≤ 16	≤ 2	≤ 2	≤ 0.25	≤ 0.25
	36D2	≤ 4	≤ 1	≤ 1	≤ 0.12	≤ 0.25	≤ 1	≤ 1	≤ 2	≤ 1	≤ 1	1	≤ 1	≤ 0.25	≤ 0.5	≤ 2	≤ 8	≤ 16	8	4	0.5	≤ 0.25
	36C3	≤ 4	≤ 1	≤ 1	≤ 0.12	≤ 0.25	≤ 1	≤ 1	≤ 2	≤ 1	≤ 1	≤ 0.25	≤ 1	≤ 0.25	≤ 0.5	≤ 2	≤ 8	≤ 16	≤ 2	≤ 2	≤ 0.25	≤ 0.25
	37P2	≤ 4	≤ 1	≤ 1	≤ 0.12	≤ 0.25	≤ 1	≤ 1	≤ 2	≤ 1	≤ 1	≤ 0.25	≤ 1	≤ 0.25	≤ 0.5	≤ 2	≤ 8	≤ 16	≤ 2	≤ 2	≤ 0.25	≤ 0.25
	37D2	≤ 4	≤ 1	≤ 1	≤ 0.12	≤ 0.25	≤ 1	≤ 1	≤ 2	≤ 1	≤ 1	2	4	≤ 0.25	≤ 0.5	≤ 2	≤ 8	≤ 16	8	8	0.5	≤ 0.25
	37C3	≤ 4	≤ 1	≤ 1	≤ 0.12	≤ 0.25	≤ 1	≤ 1	≤ 2	≤ 1	≤ 1	1	2	≤ 0.25	≤ 0.5	≤ 2	≤ 8	≤ 16	8	8	≤ 0.25	≤ 0.25
	39P2	≤ 4	≤ 1	≤ 1	≤ 0.12	≤ 0.25	≤ 1	≤ 1	≤ 2	≤ 1	≤ 1	≤ 0.25	≤ 1	≤ 0.25	≤ 0.5	≤ 2	≤ 8	≤ 16	≤ 2	≤ 2	≤ 0.25	≤ 0.25
	39D2	≤ 4	≤ 1	≤ 1	≤ 0.12	≤ 0.25	≤ 1	≤ 1	≤ 2	≤ 1	2	≤ 0.25	≤ 1	≤ 0.25	≤ 0.5	≤ 2	16	32	8	8	1	≤ 0.25
	39C3	≤ 4	≤ 1	≤ 1	≤ 0.12	≤ 0.25	≤ 1	≤ 1	≤ 2	≤ 1	≤ 1	0.5	≤ 1	≤ 0.25	≤ 0.5	≤ 2	≤ 8	≤ 16	4	4	0.5	≤ 0.25
	42P2	≤ 4	≤ 1	≤ 1	≤ 0.12	≤ 0.25	≤ 1	≤ 1	≤ 2	≤ 1	≤ 1	≤ 0.25	≤ 1	≤ 0.25	≤ 0.5	≤ 2	≤ 8	≤ 16	≤ 2	≤ 2	≤ 0.25	≤ 0.25
	42D1	≤ 4	≤ 1	≤ 1	≤ 0.12	≤ 0.25	≤ 1	≤ 1	≤ 2	≤ 1	≤ 1	≤ 0.25	≤ 1	≤ 0.25	≤ 0.5	≤ 2	≤ 8	≤ 16	≤ 2	≤ 2	≤ 0.25	≤ 0.25
	42C2	≤ 4	≤ 1	≤ 1	≤ 0.12	≤ 0.25	≤ 1	≤ 1	≤ 2	≤ 1	≤ 1	1	2	≤ 0.25	≤ 0.5	≤ 2	≤ 8	≤ 16	≤ 2	≤ 2	≤ 0.25	≤ 0.25
pH 6.0	31P3	≤ 4	2	2	≤ 0.12	≤ 0.25	≤ 1	≤ 1	≤ 2	≤ 1	≤ 1	≤ 0.25	≤ 1	≤ 0.25	≤ 0.5	≤ 2	≤ 8	≤ 16	≤ 2	≤ 2	0.5	≤ 0.25
	31D1	8	4	4	0.25	≤ 0.25	≤ 1	2	≤ 2	≤ 1	≤ 1	0.5	≤ 1	0.5	≤ 0.5	≤ 2	≤ 8	≤ 16	8	≤ 2	1	1
	31D3	≤ 4	2	2	≤ 0.12	≤ 0.25	≤ 1	≤ 1	≤ 2	≤ 1	≤ 1	1	≤ 1	≤ 0.25	≤ 0.5	≤ 2	≤ 8	≤ 16	8	≤ 2	1	≤ 0.25
	31C2	≤ 4	2	2	≤ 0.12	≤ 0.25	≤ 1	≤ 1	≤ 2	≤ 1	≤ 1	> 2	> 8	0.5	≤ 0.5	≤ 2	≤ 8	≤ 16	4	≤ 2	0.5	0.5
	31D3	≤ 4	2	2	≤ 0.12	≤ 0.25	≤ 1	≤ 1	≤ 2	≤ 1	≤ 1	> 2	> 8	≤ 0.25	≤ 0.5	≤ 2	≤ 8	≤ 16	8	≤ 2	1	≤ 0.25
	32P2	32	4	4	≤ 0.12	≤ 0.25	≤ 1	≤ 1	≤ 2	≤ 1	≤ 1	≤ 0.25	≤ 1	≤ 0.25	≤ 0.5	≤ 2	≤ 8	≤ 16	≤ 2	≤ 2	0.5	≤ 0.25
	32D2	32	4	4	≤ 0.12	≤ 0.25	≤ 1	≤ 1	≤ 2	≤ 1	≤ 1	1	≤ 1	≤ 0.25	≤ 0.5	≤ 2	16	32	4	≤ 2	1	≤ 0.25
	32D3	32	4	4	≤ 0.12	≤ 0.25	≤ 1	≤ 1	≤ 2	≤ 1	≤ 1	> 2	4	≤ 0.25	≤ 0.5	≤ 2	≤ 8	≤ 16	4	≤ 2	1	0.5
	32C2	32	8	8	≤ 0.12	≤ 0.25	≤ 1	≤ 1	≤ 2	≤ 1	≤ 1	> 2	> 8	≤ 0.25	≤ 0.5	≤ 2	≤ 8	≤ 16	≤ 2	≤ 2	1	≤ 0.25
	32C3	32	4	4	≤ 0.12	≤ 0.25	≤ 1	≤ 1	≤ 2	≤ 1	≤ 1	> 2	> 8	≤ 0.25	≤ 0.5	≤ 2	≤ 8	≤ 16	≤ 2	≤ 2	0.5	≤ 0.25
	33P3	32	4	4	≤ 0.12	≤ 0.25	≤ 1	≤ 1	≤ 2	≤ 1	≤ 1	≤ 0.25	≤ 1	≤ 0.25	> 4	≤ 2	≤ 8	≤ 16	≤ 2	≤ 2	1	≤ 0.25
	33D3	> 32	> 8	8	≤ 0.12	≤ 0.25	≤ 1	≤ 1	≤ 2	≤ 1	8	2	4	≤ 0.25	> 4	≤ 2	≤ 8	32	4	≤ 2	8	≤ 0.25
	33C3	32	8	8	≤ 0.12	≤ 0.25	≤ 1	≤ 1	≤ 2	≤ 1	≤ 1	> 2	> 8	≤ 0.25	> 4	≤ 2	≤ 8	≤ 16	≤ 2	≤ 2	2	≤ 0.25
	34P2	32	> 8	8	≤ 0.12	≤ 0.25	4	≤ 1	≤ 2	≤ 1	≤ 1	≤ 0.25	2	0.5	> 4	≤ 2	≤ 8	32	≤ 2	≤ 2	2	0.5
	34D1	> 32	> 8	> 8	≤ 0.12	≤ 0.25	8	≤ 1	≤ 2	≤ 1	2	> 2	> 8	0.5	> 4	≤ 2	≤ 8	64	≤ 2	≤ 2	8	0.5
	34D3	32	> 8	8	≤ 0.12	≤ 0.25	4	≤ 1	≤ 2	≤ 1	≤ 1	> 2	> 8	0.5	> 4	≤ 2	≤ 8	64	≤ 2	≤ 2	2	0.5
	34C	32	> 8	8	≤ 0.12	≤ 0.25	4	≤ 1	≤ 2	≤ 1	8	> 2	> 8	0.5	> 4	≤ 2	32	128	4	≤ 2	8	0.5
	34C3	> 32	> 8	> 8	1	≤ 0.25	8	≤ 1	≤ 2	≤ 1	2	> 2	> 8	2	> 4	≤ 2	≤ 8	32	4	≤ 2	> 8	1
	36P2	≤ 4	≤ 1	≤ 1	≤ 0.12	≤ 0.25	≤ 1	≤ 1	≤ 2	≤ 1	≤ 1	≤ 0.25	≤ 1	≤ 0.25	≤ 0.5	≤ 2	≤ 8	≤ 16	≤ 2	≤ 2	≤ 0.25	≤ 0.25
	36D2	8	2	4	≤ 0.12	≤ 0.25	≤ 1	≤ 1	≤ 2	≤ 1	≤ 1	> 2	4	≤ 0.25	≤ 0.5	≤ 2	≤ 8	≤ 16	≤ 2	≤ 2	0.5	≤ 0.25
	36C3	≤ 4	≤ 1	≤ 1	≤ 0.12	≤ 0.25	≤ 1	≤ 1	≤ 2	≤ 1	≤ 1	1	≤ 1	≤ 0.25	≤ 0.5	≤ 2	≤ 8	≤ 16	≤ 2	≤ 2	≤ 0.25	≤ 0.25
	37P2	8	2	2	≤ 0.12	≤ 0.25	≤ 1	≤ 1	≤ 2	≤ 1	≤ 1	≤ 0.25	≤ 1	≤ 0.25	≤ 0.5	≤ 2	≤ 8	≤ 16	≤ 2	≤ 2	≤ 0.25	≤ 0.25
	37D2	16	4	4	≤ 0.12	≤ 0.25	≤ 1	≤ 1	≤ 2	≤ 1	≤ 1	≤ 0.25	≤ 1	≤ 0.25	≤ 0.5	≤ 2	≤ 8	≤ 16	4	≤ 2	0.5	≤ 0.25
	37C3	8	2	2	≤ 0.12	≤ 0.25	≤ 1	≤ 1	≤ 2	≤ 1	≤ 1	> 2	8	≤ 0.25	≤ 0.5	≤ 2	≤ 8	≤ 16	16	8	1	≤ 0.25
	39P2	≤ 4	≤ 1	≤ 1	≤ 0.12	≤ 0.25	≤ 1	≤ 1	≤ 2	≤ 1	≤ 1	≤ 0.25	≤ 1	≤ 0.25	≤ 0.5	≤ 2	≤ 8	≤ 16	≤ 2	≤ 2	≤ 0.25	≤ 0.25
	39D2	8	2	2	≤ 0.12	≤ 0.25	≤ 1	≤ 1	≤ 2	≤ 1	≤ 1	≤ 0.25	≤ 1	≤ 0.25	≤ 0.5	≤ 2	≤ 8	≤ 16	≤ 2	≤ 2	≤ 0.25	≤ 0.25
	39C3	≤ 4	≤ 1	2	≤ 0.12	≤ 0.25	≤ 1	≤ 1	≤ 2	≤ 1	≤ 1	> 2	8	≤ 0.25	≤ 0.5	≤ 2	≤ 8	≤ 16	≤ 2	≤ 2	0.5	≤ 0.25
	42P2	8	2	2	≤ 0.12	≤ 0.25	≤ 1	≤ 1	≤ 2	≤ 1	≤ 1	≤ 0.25	≤ 1	≤ 0.25	≤ 0.5	≤ 2	≤ 8	≤ 16	≤ 2	≤ 2	≤ 0.25	0.5
	42D3	8	2	2	≤ 0.12	≤ 0.25	≤ 1	≤ 1	≤ 2	≤ 1	≤ 1	≤ 0.25	≤ 1	≤ 0.25	≤ 0.5	≤ 2	≤ 8	≤ 16	≤ 2	≤ 2	0.5	≤ 0.25
	42C2	8	2	2	≤ 0.12	≤ 0.25	≤ 1	≤ 1	≤ 2	≤ 1	≤ 1	> 2	4	≤ 0.25	≤ 0.5	≤ 2	≤ 8	≤ 16	≤ 2	≤ 2	0.5	≤ 0.25

AMI = Amikacin, GEN = Gentamicin, TOB = Tobramycin, DOR = Doripenem, ETP = Ertapenem, IMI = Imipenem, MERO = Meropenem, FEP = Cefepime, FOT = Cefotaxime, TAZ = Ceftazidime, CIP = Ciproflxacin, LEVO = Levofloxacin, COL = Colistin, SXT = Trimethoprim/sulfamethoxazole, AZT = Aztreonam, P/T4 = Piperacillin/Tazobactam constant 4, TIM2 = Ticarcillin/clavulanic acid constant 2, DOX = Doxycycline, MIN = Minocycline, TGC = Tigecycline, POL = Polymyxin B
