# Supplementary material for: Resistance development in Escherichia coli to delafloxacin at pHs 6.0 and 7.3 compared to ciprofloxacin
Source: Antimicrob Agents Chemother. 2023 Oct 26;67(11):e01625-22. doi: 10.1128/aac.01625-22 (PMC10649057; doi:10.1128/aac.01625-22)
Supplement: Table S2 — MIC fold-reduction of parental isolates and derivatives after the addition of PAbN. [file aac.01625-22-s0009.pdf]

Table S2. MIC fold-reductions of Parental Isolates and Derivatives after Addition of PAßN

ID of derivative	Culturing conditions		Parental strain		Parental strain + PAßN		Parental Ratio*		Derivative strain		Derivative strain + PAßN		Derivative Ratio**	
	Antibiotic	pH	DLX MIC	CIP MIC	DLX MIC	CIP MIC	DLX MIC	CIP MIC	DLX MIC	CIP MIC	DLX MIC	CIP MIC	DLX MIC	CIP MIC
31D1	DLX	6.0	0.016	0.064	0.002	0.064	8	1	0.5	0.75	0.008	0.125	62.5	6
31D3	DLX	6.0	0.016	0.064	0.002	0.064	8	1	1	1	0.032	0.5	32	2
32D2	DLX	6.0	0.016	0.064	0.002	0.064	8	1	0.125	0.25	0.004	0.125	32	2
32D3	DLX	6.0	0.016	0.064	0.002	0.064	8	1	2	8	0.032	4	64	2
33D3	DLX	6.0	0.032	0.125	0.002	0.125	16	1	0.125	0.5	0.004	0.25	32	2
34D1	DLX	6.0	0.008	0.032	0.002	0.032	4	1	0.25	0.25	0.004	0.125	64	2
34D3	DLX	6.0	0.008	0.032	0.002	0.032	4	1	1	4	0.032	0.5	32	8
36D2	DLX	6.0	0.008	0.016	0.00025	0.016	32	1	0.5	1	0.004	0.5	125	2
37D2	DLX	6.0	0.016	0.032	0.002	0.032	8	1	0.125	0.125	0.008	0.032	16	4
39D2	DLX	6.0	0.004	0.064	0.001	0.064	4	1	0.032	0.064	0.002	0.032	16	2
42D3	DLX	6.0	0.016	0.064	0.001	0.064	16	1	0.125	0.25	0.008	0.032	16	8
31D1	DLX	7.3	0.047	0.008	0.016	0.008	3	1	4	0.125	0.25	0.006	16	21
31D3	DLX	7.3	0.047	0.008	0.016	0.008	3	1	2	0.25	0.125	0.032	16	8
32D2	DLX	7.3	0.064	0.008	0.008	0.016	8	0.5	1	0.125	0.064	0.125	16	1
32D3	DLX	7.3	0.064	0.008	0.008	0.016	8	0.5	16	4	4	2	4	2
33D1	DLX	7.3	0.064	0.016	0.004	0.016	16	1	16	8	1	2	16	4
34D2	DLX	7.3	0.064	0.016	0.008	0.016	8	1	1	0.25	0.064	0.25	16	1
34D3	DLX	7.3	0.064	0.016	0.008	0.016	8	1	32	2	1	2	32	1
36D1	DLX	7.3	0.016	0.008	0.002	0.008	8	1	0.25	0.032	0.125	0.016	2	2
36D2	DLX	7.3	0.016	0.008	0.002	0.008	8	1	2	0.5	0.5	0.5	4	1
37D2	DLX	7.3	0.064	0.016	0.016	0.016	4	1	4	2	1	1	4	2
39D2	DLX	7.3	0.032	0.008	0.008	0.016	4	0.5	1	0.125	0.5	0.064	2	2
42D1	DLX	7.3	0.032	0.008	0.008	0.016	4	0.5	0.25	0.032	0.016	0.032	16	1
31C2	CIP	6.0	0.016	0.064	0.002	0.064	8	1	0.25	8	0.008	8	32	1
31C3	CIP	6.0	0.016	0.064	0.002	0.064	8	1	2	16	0.064	16	32	1
32C2	CIP	6.0	0.016	0.064	0.002	0.064	8	1	0.125	32	0.002	16	64	2
32C3	CIP	6.0	0.016	0.064	0.002	0.064	8	1	2	32	0.032	8	64	4
33C3	CIP	6.0	0.032	0.125	0.002	0.125	16	1	1	64	0.032	64	32	1
34C2	CIP	6.0	0.008	0.032	0.002	0.032	4	1	0.25	8	0.004	6	64	1.3
34C3	CIP	6.0	0.008	0.032	0.002	0.032	4	1	2	32	0.064	16	32	2
36C3	CIP	6.0	0.008	0.016	0.00025	0.016	32	1	0.032	0.25	0.002	0.25	16	1
37C1	CIP	6.0	0.016	0.032	0.002	0.032	8	1	4	64	0.25	64	16	1
37C3	CIP	6.0	0.016	0.032	0.002	0.032	8	1	0.25	4	0.032	2	8	2
39C3	CIP	6.0	0.004	0.064	0.001	0.064	4	1	0.064	8	0.002	4	32	2
42C2	CIP	6.0	0.016	0.064	0.001	0.064	16	1	0.125	4	0.004	2	32	2
31C3	CIP	7.3	0.047	0.008	0.016	0.008	3	1	4	2	0.125	1	32	2
32C1	CIP	7.3	0.064	0.008	0.008	0.016	8	0.5	0.5	1	0.064	0.5	8	2
32C2	CIP	7.3	0.064	0.008	0.008	0.016	8	0.5	0.5	0.25	0.032	0.25	16	1
33C1	CIP	7.3	0.064	0.016	0.004	0.016	16	1	4	32	0.25	32	16	1
33C3	CIP	7.3	0.064	0.016	0.004	0.016	16	1	0.5	1	0.032	0.5	16	2
34C1	CIP	7.3	0.064	0.016	0.008	0.016	8	1	1	1	0.064	0.5	16	2
34C2	CIP	7.3	0.064	0.016	0.008	0.016	8	1	0.5	0.5	0.016	0.25	32	2
36C3	CIP	7.3	0.016	0.008	0.002	0.008	8	1	0.125	0.125	0.004	0.125	32	1

37C3	CIP	7.3	0.064	0.016	0.016	0.016	4	1	1	1	0.25	0.5	4	2
39C3	CIP	7.3	0.032	0.008	0.008	0.016	4	0.5	0.25	0.5	0.016	0.25	16	2
42C2	CIP	7.3	0.032	0.008	0.008	0.016	4	0.5	1	2	0.5	1	2	2

*x-fold reduction of MIC after adding PAßN in parentals compared to the parentals without PAßN; ** x-fold reduction of MIC after adding PAßN in derivatives compared to the derivatives without PAßN
