# Supplementary material for: Resistance development in Escherichia coli to delafloxacin at pHs 6.0 and 7.3 compared to ciprofloxacin
Source: Antimicrob Agents Chemother. 2023 Oct 26;67(11):e01625-22. doi: 10.1128/aac.01625-22 (PMC10649057; doi:10.1128/aac.01625-22)
Supplement: Table S3 — Overview of all mutations in derivatives and the effect of PaBN. [file aac.01625-22-s0010.pdf]

Table S3. List of Parental Isolates, their evolved Derivatives, MICs with and without pAβN and detected Mutations

Parental	DLX MIC (parental)	CIP MIC (parental)	Derivative	pH	Antibiotic “challenge»	DLX MIC (Derivative)	CIP MIC (Derivative)	MIC reduction fold DLX with PaβN*	MIC reduction fold CIP with PaβN*	Gene	Nucleotide	Aminoacid	Gene function	source
31	0.016	0.064	31D1	6.0	DLX	0.5	0.5	4	4	marR	G205A	G69R	Transcriptional repressor of marRAB operon, regulation of AcrAB MDR efflux	(1)
										emrR	G329A	R110H	transcriptional repressor of emrRAB operon, regulation of EmrAB MDR efflux	(1)
										rpoB	C451A	R151S	encodes the β subunit of bacterial RNA polymerase	(2)
31	0.016	0.064	31D3	6.0	DLX	1	1	4	2	gyrA	G1835T	R612L	DNA gyrase	(3)
										marR	C217T	R73C	Transcriptional repressor of marRAB operon, regulation of AcrAB MDR efflux	(1)
32	0.016	0.064	32D2	6.0	DLX	0.125	0.25	4	2	gyrA	C2156A	T719N	DNA gyrase	(3)
										marR	C217T	R73C	Transcriptional repressor of marRAB operon, regulation of AcrAB MDR efflux	(1)
										marR	AT220-	M74G	Transcriptional repressor of marRAB operon, regulation of AcrAB MDR efflux	(1)
										rutA	G826A	E276K	Pyrimidine monooxygenase	(4)
32	0.016	0.064	32D3	6.0	DLX	2	8	4	2	gyrA	C248T	S83L	DNA gyrase	(3)
										emrR	C251A	S84Y	transcriptional repressor of emrRAB operon, regulation of EmrAB MDR efflux	(1)
										parC	C1690A	R564S	DNA topoisomerase IV	(3)
33	0.032	0.125	33D3	6.0	DLX	0.125	0.5	2	2	gyrA	C1834G	R612G	DNA gyrase	(3)
										ynfF	G1474C	D492H	Anaerobic dimethyl sulfoxide reductase	(5)
34	0.008	0.032	34D1	6.0	DLX	0.25	0.25	8	2	gyrA	G1846A	G616S	DNA gyrase	(3)
										marR	G206A	G69E	Transcriptional repressor of marRAB operon, regulation of AcrAB MDR efflux	(1)
										mdtC	G1894A	V632I	Multidrug resistance protein, belonging to MdtABC-TolC (RND) efflux	(6)
										emrR	C331T	R111C	transcriptional repressor of emrRAB operon, regulation of EmrAB MDR efflux	(1)
										tfaQ	C251A	A84E	Prophage tail fiber assembly protein homolog	(7)
										yfcI	G834T	E278D	Transposase_31 family protein	(8)
34	0.008	0.032	34D3	6.0	DLX	1	4	8	8	gyrA	C248T	S83L	DNA gyrase	(3)
										mdtC	G1894A	V632I	Multidrug resistance protein, belonging to MdtABC-TolC (RND) efflux	(7)
										emrR	C412A	Q138K	transcriptional repressor of emrRAB operon, regulation of EmrAB MDR efflux	(1)
										parE	C1831T	R611C	DNA topoisomerase IV	(3)
										tfaQ	C251A	A84E	Prophage tail fiber assembly protein homolog	(7)
										yfcI	G834T	E278D	Transposase_31 family protein	(8)
36	0.008	0.016	36D2	6.0	DLX	0.5	1	2	2	gyrA	C248T	S83L	DNA gyrase	(3)
										ParE	G1832T	R611L	DNA topoisomerase IV	(3)
37	0.016	0.032	37D2	6.0	DLX	0.125	0.125	2	4	marR	A214C	T72P	Transcriptional repressor of marRAB operon, regulation of AcrAB MDR efflux	(1)
										emrR	A326C	D109A	transcriptional repressor of emrRAB operon, regulation of EmrAB MDR efflux	(1)
39	0.004	0.064	39D2	6.0	DLX	0.032	0.064	4	2	emrR	C331A	R111S	transcriptional repressor of emrRAB operon, regulation of EmrAB MDR efflux	(1)
31	0.047	0.008	31D1	7.3	DLX	4	0.125	1	2	gyrA	C1832T	T611I	DNA gyrase	(3)
31	0.047	0.008	31D3	7.3	DLX	2	0.25	1	7	gyrA	C1841T	A614V	DNA gyrase	(3)
										marR	T233C	L78P	Transcriptional repressor of marRAB operon, regulation of AcrAB MDR efflux	(1)
										emrR	T476C	L159P	transcriptional repressor of emrRAB operon, regulation of EmrAB MDR efflux	(1)
32	0.064	0.008	32D3	7.3	DLX	16	4	1	4	gyrA	C248T	S83L	DNA gyrase	(3)
										marR	A115G	T39A	Transcriptional repressor of marRAB operon, regulation of AcrAB MDR efflux	(1)
										emrR	T418A	W140R	transcriptional repressor of emrRAB operon, regulation of EmrAB MDR efflux	(1)
										parE	C1193T	P398L	DNA topoisomerase IV	(3)
										rpoB	T3994C	S1332P	encodes the β subunit of bacterial RNA polymerase	(2)
										uspG	A239C	D80A	universal stress protein	(9)
33	0.064	0.016	33D1	7.3	DLX	16	8	1	4	aceB	A1090G	T364A	Malate synthase	(10)
										asmA	A1501G	T501A	Suppressor of ompf assembly mutants	(11)
										cynT	G61A	A21T	Carbonic anhydrase	(12)
										deoC	A268G	T90A	Deoxyribose-phosphate aldolase	(13)
										dosC	A303G	I101M	Diguanylate cyclase	(14)
										dsbD	A415G	S139G	cytoplasmic membrane protein	(15)
										galS	A142G	N48D	HTH-type transcriptional regulator, mgl repressor (galactose transport/utilization)	(16)
										gcd	T1547C	V516A	Quinoprotein glucose dehydrogenase	(17)
										gcd	T248C	V83A	Quinoprotein glucose dehydrogenase	(17)
										glcF	G776A	F259E	Glycolate oxidase iron-sulfur subunit	(18)
										gpmM	T1076C	L359P	Phosphoglycerate mutase	(19)
										gshA	A64G	I22V	Glutamate-cysteine ligase	(20)
										gyrA	C248T	S83L	DNA gyrase	(3)
										idi	A206G	H69R	Isopentenyl-diphosphate Delta-isomerase	(7)
										lacI	T356C	V119A	Lactose operon repressor	(21)
										lon	A1345G	N449D	ATP-dependent serine protease	(7)
										mdtC	C547G	P183A	Multidrug resistance protein, belonging to MdtABC-TolC (RND) efflux	(6)
										mutS	T89A	F30Y	DNA mismatch repair protein	(22)
										mutS	A92T	Y31F	DNA mismatch repair protein	(22)
										oxyR	A530G	N177S	positive regulator of hydrogen peroxide-inducible genes	(23)
										parC	A418G	T140A	DNA topoisomerase IV	(3)
										pflB	G1440A	Mt480I	pyruvate formate-lyase	(7)
										pgpB	C224T	A75V	P-glycoprotein	(24)
										ptrB	A1478G	Y493C	oligopeptidase	(25)
										pykF	T437C	V146A	Pyruvate kinase I	(26)
										soxR	A175G	I59V	sensory protein that upregulates soxS expression, leads to acrAB efflux expression	(27)
										soxR	C437T	A146V	sensory protein that upregulates soxS expression, leads to acrAB efflux expression	(27)
										xanP	T1136C	V379A	Xanthine permease	(7)
										yciM	C1127A	A376D	regulation of lipopolysaccharide synthesis	(28)
										ycjM	A335G	E112G	regulation of lipopolysaccharide synthesis	(28)
										ydhK	C1508T	A503V	uncharacterized member of the Aromatic Acid Exporter (ArAE) family	(7)
										ydhU	T412C	Y138H	uncharacterized member of the Aromatic Acid Exporter (ArAE) family	(7)
										yeiL	T287C	L96P	member of the CRP-FNR family	(29)
										ygcN	T1061C	V354A	Electron transfer flavoprotein-quinone oxidoreductase	(7)
										yibH	A389G	N130S	HlyD family secretion protein	(7)
34	0.064	0.016	34D3	7.3	DLX	32	2	1	4	glnA	G154A	G52S	Metabolic regulation	(30)
										gyrA	A260G	D87G	DNA gyrase	(3)
										gyrA	C248T	S83L	DNA gyrase	(3)
										yfcI	G834T	E278D	Transposase_31 family protein	(8)
										mdtC	G1894A	V632I	Multidrug resistance protein, belonging to MdtABC-TolC (RND) efflux	(6)
										emrR	T452A	L151H	transcriptional repressor of emrRAB operon, regulation of EmrAB MDR efflux	(1)
										yehU	A5G	Y2C	Histidine kinase	(33)
										yehU	A5C	Y2S		
										parC	C350T	A117V	DNA topoisomerase IV	(3)

Parental	DLX MIC (parental)	CIP MIC (parental)	Derivative	pH	Antibiotic “challenge»	DLX MIC (Derivative)	CIP MIC (Derivative)	MIC reduction fold DLX with PaβN*	MIC reduction fold CIP with PaβN*	Gene	Nucleotide	Aminoacid	Gene function	source
36	0.016	0.008	36D2	7.3	DLX	2	0.5	0.5	1	gyrA	C248T	S83L	DNA gyrase	(3)
										emrR	A326G	D109G	transcriptional repressor of emrRAB operon, regulation of EmrAB MDR efflux	(1)
										parC	C350T	A117V	DNA topoisomerase IV	(3)
42	0.032	0.016	42D1	7.3	DLX	0.25	0.032	4	0.5	gyrA	A1814G	Y605C	DNA gyrase	(3)
										emrR	T191C	L64P	transcriptional repressor of emrRAB operon, regulation of EmrAB MDR efflux	(1)
31	0.016	0.064	31C2	6.0	CIP	0.25	2	4	2	envZ	G719A	G240E	histidine kinase/phosphatase, regulation of OmpR, indirect regulation of OmpF and C	(34)
										gyrA	A260G	D87G	DNA gyrase	(3)
										gyrB	C1391T	S464F	DNA gyrase	(3)
										parC	C350A	A117E	DNA topoisomerase IV	(3)
										soxR	C58T	R20C	sensory protein that upregulates soxS expression, leads to acrAB efflux expression	(27)
31	0.016	0.064	31C3	6.0	CIP	2	16	4	2	gyrA	C248T	S83L	DNA gyrase	(3)
										marR	GAA253-	E85I	Transcriptional repressor of marRAB operon, regulation of AcrAB MDR efflux	(1)
										marR	G258T	R86S	Transcriptional repressor of marRAB operon, regulation of AcrAB MDR efflux	(1)
										marR	T260G	L87W	Transcriptional repressor of marRAB operon, regulation of AcrAB MDR efflux	(1)
										pgrR	G167A	R56H	Regulator PgrR for switch control of peptidoglycan recycling	(35)
32	0.016	0.064	32C2	6.0	CIP	0.125	32	4	2	gyrA	G259T	D87Y	DNA gyrase	(3)
										gyrB	C1391A	S464Y	DNA gyrase	(3)
										parE	G1378A	E460K	DNA topoisomerase IV	(3)
32	0.016	0.064	32C3	6.0	CIP	2	32	2	2	gyrA	C248T	S83L	DNA gyrase	(3)
										gyrA	G259T	D87Y	DNA gyrase	(3)
33	0.032	0.125	33C3	6.0	CIP	1	64	2	1	gyrA	A260G	D87G	DNA gyrase	(3)
										gyrA	C248T	S83L	DNA gyrase	(3)
										parC	G232T	G78C	DNA topoisomerase IV	(3)
										parE	C1373G	S458W	DNA topoisomerase IV	(3)
										sgrR	C1652T	P551L	Transcriptional activator of sgrs under glucose-phosphate stress	(36)
										yehU	T569A	F190Y	Histidine kinase	(33)
										yehU	CT565-	L189A	Histidine kinase	(33)
										ynfF	G1474C	D492H	Anaerobic dimethyl sulfoxide reductase	(5)
34	0.008	0.032	34C2	6.0	CIP	0.25	8	8	1	aroK	T71C	L24S	Shikimate kinase	(7)
										envZ	G326T	S109I	histidine kinase/phosphatase, regulation of OmpR, indirect regulation of OmpF and C	(34)
										gyrA	A260C	D87A	DNA gyrase	(3)
										rpoB	C2280G	N760K	encodes the β subunit of bacterial RNA polymerase	(2)
										yfcI	G834T	E278D	Transposase_31 family protein	(8)
34	0.008	0.032	34C3	6.0	CIP	2	32	7	2	gyrA	C248T	S83L	DNA gyrase	(3)
										mdtC	G1894A	V632I	Multidrug resistance protein, belonging to MdtABC-TolC (RND) efflux	(6)
										tfaQ	C251A	A84E	Prophage tail fiber assembly protein homolog	(7)
										yfcI	G834T	E278D	Transposase_31 family protein	(8)
36	0.008	0.016	36C3	6.0	CIP	0.032	0.25	5	1	envZ	T31C	S11P	histidine kinase/phosphatase, regulation of OmpR, indirect regulation of OmpF and C	(34)
										gyrA	G259T	D87Y	DNA gyrase	(3)
37	0.016	0.032	37C1	6.0	CIP	4	64	2	1	gntK	A355G	S119G	Gluconokinase	(7)
										gyrA	C248T	S83L	DNA gyrase	(3)
										gyrA	G259T	D87Y	DNA gyrase	(3)
										mutS	T1349A	L450Q	DNA mismatch repair protein	(22)
										ParC	C240A	S80R	DNA topoisomerase IV	(3)
										parE	A1259G	D420G	DNA topoisomerase IV	(3)
										pepD	A1454G	K485R	dipeptidase	(7)
										proP	A496T	S166C	Proline-betaine-transporter	(37)
										rcnA	G721A	G241R	Nickel/cobalt efflux system	(7)
										rep	C1064T	S355L	ATP-dependent rep helicase	(38)
										slyA	C256T	R86W	Transcriptional regulator of the Capsule Gene Cluster	(39)
										ydjN	C1097T	P366L	S-sulfocysteine transporter	(40)
										yeaY	C140T	A47V	Putative lipoprotein	(7)
										yfhM	A988G	T330A	polypeptide α2-macroglobulin.	(7)
										yicR	G158A	G53D	Radc-like jab domain-containing protein	(7)
37	0.016	0.032	37C3	6.0	CIP	0.25	4	1	2	gyrA	G259T	D87Y	DNA gyrase	(3)
										marR	A214C	T72P	Transcriptional repressor of marRAB operon, regulation of AcrAB MDR efflux	(1)
										yfgF	G994A	V332M	anaerobic cyclic di-GMP phosphodiesterase	(41)
39	0.004	0.064	39C3	6.0	CIP	0.064	8	8	2	envZ	T722G	V241G	histidine kinase/phosphatase, regulation of OmpR, indirect regulation of OmpF and C	(34)
										gyrA	A260G	D87G	DNA gyrase	(3)
										gyrA	G250C	A84P	DNA gyrase	(3)
										parE	A1330T	I444F	DNA topoisomerase IV	(3)
42	0.016	0.064	42C2	6.0	CIP	0.125	4	2	2	envZ	A698G	D233G	histidine kinase/phosphatase, regulation of OmpR, indirect regulation of OmpF and C	(34)
										gyrA	G259A	D87N	DNA gyrase	(3)
										yceJ	T524G	L175R	Putative cytochrome	(7)
31	0.047	0.008	31C3	7.3	CIP	4	2	2	2	gyrA	A260G	D87G	DNA gyrase	(3)
										gyrA	C248T	S83L	DNA gyrase	(3)
										ydhK	A505C	I169L	uncharacterized member of the Aromatic Acid Exporter (ArAE) family	(7)
32	0.064	0.008	32C1	7.3	CIP	0.5	1	1	4	acrR	T532A	W178R	transcriptional repressor of AcrAB-TolC efflux pump	(42)
										emrB	AT454-	I152D	MFS transporterof EmrAB-TolC efflux pump	(43)
										emrB	T467A	I156N	MFS transporterof EmrAB-TolC efflux pump	(43)
										gyrA	A260G	D87G	DNA gyrase	(3)
										rpoC	G244A	G82S	encodes the β subunit of bacterial RNA polymerase	(2)
32	0.064	0.008	32C2	7.3	CIP	0.5	0.25	2	2	gyrA	A260G	D87G	DNA gyrase	(3)
										soxR	G362A	G121D	sensory protein that upregulates soxS expression, leads to acrAB efflux expression	(27)

Parental	DLX MIC (parental)	CIP MIC (parental)	Derivative	pH	Antibiotic “challenge»	DLX MIC (Derivative)	CIP MIC (Derivative)	MIC reduction fold DLX with PaβN*	MIC reduction fold CIP with PaβN*	Gene	Nucleotide	Aminoacid	Gene function	source
33	0.064	0.016	33C1	7.3	CIP	4	32	1	1	actP	G419A	R140H	Cation/acetate smporter	(7)
										adeD	G959A	S320NAsn	Adenine deaminase	(7)
										arnA	T1315C	C439R	Bifunctional polymyxin resistance protein	(44)
										bamE	C88T	P30S	Outer membrane protein assembly factor	(7)
										chaA	C644T	P215L	sodium ion extrusion	(45)
										cstA	G370A	V124M	Pyruvate transporter	(46)
										damX	A310G	M104V	Inner membrane protein	(47)
										dosC	G1243A	A415T	Diguanylate cyclase	(14)
										emrK	G818A	G273D	Multidrug resistance efflux pump membrane fusion protein	(48)
										fimD	A784G	T262A	Outer membrane usher protein, surface localization of type 1 fimbriae	(49)
										flgL	A713G	N238S	Flagellar hook-associated protein	(7)
										gyrA	A260G	D87G	DNA gyrase	(3)
										icd	C937A	L313M	Isocitrate dehydrogenase	(7)
										mdlA	A1271G	D424G	encodes protein related to the ATP-binding component of a MDR transport resistance	(50)
										mdtM	A292G	T98A	Major facilitator superfamily multidrug transporter	(51)
										nfuA	A32G	H11R	encoding Fe/S biogenesis assisting factors	(7)
										nlpD	C599A	A200D	Lipoprotein involved in cell wall remodeling	(52)
										parC	C1846T	R616C	DNA topoisomerase IV	(3)
										parC	G239T	S80I	DNA topoisomerase IV	(3)
										ppdA	C329T	S110L	prepilin peptidase-dependent protein A	(7)
										ptrA	A2632G	K878E	oligopeptidase	(25)
										queC	T391C	Y131H	7-cyano-7-deazaguanine synthase	(7)
										sfmD	T2047C	S683P	Outer membrane usher protein	(7)
										trkH	T226C	F76L	Potassium transporter	(7)
										tyrP	A43G	T15A	Tyrosine-specific transport system	(53)
										uvrD	A353G	D118G	Helicase II, ATP dependent ssDNA translocase	(7)
										yciV	G112A	G38S	3',5'-nucleoside bisphosphate phosphatase	(7)
										yggW	C962T	A321V	Putative coproporphyrinogen-iii oxidase-like protein	(7)
										yjeI	A113G	Q38R	lipoprotein	(54)
										yjjB	T278C	V93A	Probable succinate transporter subunit	(7)
										yqjA	A508G	T170A	Member of DedA/Tvp38 Membrane Protein, Putative Osmosensing Transporter	(55)
33	0.064	0.016	33C3	7.3	CIP	0.75	1	1	1	acrR	A426C	E142D	transcriptional repressor of AcrAB-TolC efflux pump	(42)
										gyrA	A260G	D87G	DNA gyrase	(3)
34	0.064	0.016	34C1	7.3	CIP	1	1	2	2	gyrA	G259T	D87Y	DNA gyrase	(3)
										marR	G206A	G69E	Transcriptional repressor of marRAB operon, regulation of AcrAB MDR efflux	(1)
										mdtC	G1894A	V632I	Multidrug resistance protein, belonging to MdtABC-TolC (RND) efflux	(6)
										tfaQ	C251A	A84E	Prophage tail fiber assembly protein homolog	(7)
										yfcI	G834T	E278D	Transposase_31 family protein	(8)
34	0.064	0.016	34C2	7.3	CIP	0.5	0.5	4	2	envZ	T257G	I86S	histidine kinase/phosphatase, regulation of OmpR, indirect regulation of OmpF and C	(34)
										gyrA	G259T	D87Y	DNA gyrase	(3)
										hyi	G59T	R20L	Hydroxypyruvate isomerase	(7)
										mdtC	G1894A	V632I	Multidrug resistance protein, belonging to MdtABC-TolC (RND) efflux	(6)
										emrR	A166G	T56A	transcriptional repressor of emrRAB operon, regulation of EmrAB MDR efflux	(1)
										emrR	T172A	F58I	transcriptional repressor of emrRAB operon, regulation of EmrAB MDR efflux	(1)
										tfaQ	C251A	A84E	Prophage tail fiber assembly protein homolog	(7)
										yfcI	G834T	E278D	Transposase_31 family protein	(8)
36	0.016	0.008	36C3	7.3	CIP	0.125	0.125	4	1	acrR	T86G	V29G	transcriptional repressor of AcrAB-TolC efflux pump	(42)
										envZ	C692T	A231V	histidine kinase/phosphatase, regulation of OmpR, indirect regulation of OmpF and C	(34)
										gyrA	A260G	D87G	DNA gyrase	(3)
										yggF	T815C	V272A	Fructose-1,6-bisphosphatase	(7)
37	0.064	0.016	37C3	7.3	CIP	1	1	1	2	etk	C1480T	H494Y	Escherichia coli Tyr kinase	(56)
										gyrA	C248T	S83L	DNA gyrase	(3)
										rstA	A545G	D182G	two-component response regulator, plays role in virulence	(57)
										sucA	C1448A	P483Q	2-oxoglutarate dehydrogenase	(7)
										yaeR	G155C	G52A	Uncharacterized protein	(7)
39	0.032	0.016	39C3	7.3	CIP	0.25	0.5	3	1	gyrA	A260G	D87G	DNA gyrase	(3)
42	0.032	0.016	42C2	7.3	CIP	1	2	1	1	gyrA	C248T	S83L	DNA gyrase	(3)

* MIC reduction fold with PaβN is calculated as MIC reduction in the derivatives divided by the MIC reduction in the parental isolates after adding PaβN

References:

1. Beggs GA, Brennan RG, Arshad M. MarR family proteins are important regulators of clinically relevant antibiotic resistance. Protein Sci Publ Protein Soc. März 2020;29(3):647–53.

2. Garibyan L. Use of the rpoB gene to determine the specificity of base substitution mutations on the Escherichia coli chromosome. DNA Repair. 13. Mai 2003;2(5):593–608.

3. Drlica K, Hiasa H, Kerns R, Malik M, Mustaev A, Zhao X. Quinolones: Action and Resistance Updated. Curr Top Med Chem. 1. August 2009;9(11):981–98.

4. Kim KS, Pelton JG, Inwood WB, Andersen U, Kustu S, Wemmer DE. The Rut Pathway for Pyrimidine Degradation: Novel Chemistry and Toxicity Problems. J Bacteriol. 15. August 2010;192(16):4089–102.

5. Lubitz SP, Weiner JH. The Escherichia coli ynfEFGHI operon encodes polypeptides which are paralogues of dimethyl sulfoxide reductase (DmsABC). Arch Biochem Biophys. Oktober 2003;418(2):205–16.

6. Kim HS, Nagore D, Nikaido H. Multidrug Efflux Pump MdtBC of *Escherichia coli* Is Active Only as a B $_2$ C Heterotrimer. J Bacteriol. März 2010;192(5):1377–86.

7. Blattner FR, Plunkett G, Bloch CA, Perna NT, Burland V, Riley M, u. a. The Complete Genome Sequence of *Escherichia coli* K-12. Science. 5. September 1997;277(5331):1453–62.

8. Kingston AW, Ponkratz C, Raleigh EA. Rpn (YhgA-Like) Proteins of Escherichia coli K-12 and Their Contribution to RecA-Independent Horizontal Transfer. Gourse RL, Herausgeber. J Bacteriol [Internet]. April 2017 [zitiert 4. April 2023];199(7). Verfügbar unter: https://journals.asm.org/doi/10.1128/JB.00787-16

9. Weber A, Jung K. Biochemical Properties of UspG, a Universal Stress Protein of *Escherichia coli*. Biochemistry. 1. Februar 2006;45(6):1620–8.

10. Maloy SR, Nunn WD. Genetic regulation of the glyoxylate shunt in Escherichia coli K-12. J Bacteriol. Januar 1982;149(1):173–80.

11. Deng M, Misra R. Examination of AsmA and its effect on the assembly of Escherichia coli outer membrane proteins. Mol Microbiol. August 1996;21(3):605–12.

12. Guilloton MB, Korte JJ, Lamblin AF, Fuchs JA, Anderson PM. Carbonic anhydrase in Escherichia coli. A product of the cyn operon. J Biol Chem. 25. Februar 1992;267(6):3731–4.

13. Valentin-Hansen P, Boëtius F, Hammer-Jespersen K, Svendsen I. The Primary Structure of *Escherichia coli* K12 2-Deoxyribose 5-Phosphate Aldolase: Nucleotide Sequence of the deoC Gene and the Amino Acid Sequence of the Enzyme. Eur J Biochem. Juli 1982;125(3):561–6.

14. Gilles-Gonzalez MA, Sousa EHS. Escherichia coli DosC and DosP: a role of c-di-GMP in compartmentalized sensing by degradosomes. In: Advances in Microbial Physiology [Internet]. Elsevier; 2019 [zitiert 4. April 2023]. S. 53–67. Verfügbar unter: https://linkinghub.elsevier.com/retrieve/pii/S0065291119300220

15. Stewart EJ. Six conserved cysteines of the membrane protein DsbD are required for the transfer of electrons from the cytoplasm to the periplasm of Escherichia coli. EMBO J. 1. November 1999;18(21):5963–71.

16. Weickert MJ, Adhya S. Isorepressor of the gal regulon in Escherichia coli. J Mol Biol. Juli 1992;226(1):69–83.

17. Yamada M, Asaoka S, Saier MH, Yamada Y. Characterization of the gcd gene from Escherichia coli K-12 W3110 and regulation of its expression. J Bacteriol. Januar 1993;175(2):568–71.

18. Pellicer MT, Badía J, Aguilar J, Baldomà L. glc locus of Escherichia coli: characterization of genes encoding the subunits of glycolate oxidase and the glc regulator protein. J Bacteriol. April 1996;178(7):2051–9.

19. Pan Q, Li Z, Ju X, Hou C, Xiao Y, Shi R, u. a. *Escherichia coli* segments its controls on carbon-dependent gene expression into global and specific regulations. Microb Biotechnol. Mai 2021;14(3):1084–106.

20. Apontoweil P, Berends W. Mapping of gshA, a gene for the biosynthesis of glutathione in Eschericha coli K12. Mol Gen Genet MGG. Juni 1975;141(2):91–5.

21. Semsey S, Jauffred L, Csiszovszki Z, Erdossy J, Steger V, Hansen S, u. a. The effect of LacI autoregulation on the performance of the lactose utilization system in Escherichia coli. Nucleic Acids Res. 1. Juli 2013;41(13):6381–90.

22. Acharya S, Foster PL, Brooks P, Fishel R. The Coordinated Functions of the E. coli MutS and MutL Proteins in Mismatch Repair. Mol Cell. Juli 2003;12(1):233–46.

23. Christman MF, Storz G, Ames BN. OxyR, a positive regulator of hydrogen peroxide-inducible genes in Escherichia coli and Salmonella typhimurium, is homologous to a family of bacterial regulatory proteins. Proc Natl Acad Sci U S A. Mai 1989;86(10):3484–8.

24. Funk CR, Zimniak L, Dowhan W. The pgpA and pgpB genes of Escherichia coli are not essential: evidence for a third phosphatidylglycerophosphate phosphatase. J Bacteriol. Januar 1992;174(1):205–13.

25. Beck HJ, Janssen GR. Novel Translation Initiation Regulation Mechanism in Escherichia coli ptrB Mediated by a 5′-Terminal AUG. Henkin TM, Herausgeber. J Bacteriol [Internet]. 15. Juli 2017 [zitiert 4. April 2023];199(14). Verfügbar unter: https://journals.asm.org/doi/10.1128/JB.00091-17

26. Siddiquee KAZ, Arauzo-Bravo MJ, Shimizu K. Effect of a pyruvate kinase (pykF-gene) knockout mutation on the control of gene expression and metabolic fluxes in Escherichia coli. FEMS Microbiol Lett. 1. Juni 2004;235(1):25–33.

27. Koutsolioutsou A, Peña-Llopis S, Demple B. Constitutive *soxR* Mutations Contribute to Multiple-Antibiotic Resistance in Clinical *Escherichia coli* Isolates. Antimicrob Agents Chemother. Juli 2005;49(7):2746–52.

28. Mahalakshmi S, Sunayana MR, SaiSree L, Reddy M. yciM is an essential gene required for regulation of lipopolysaccharide synthesis in Escherichia coli. Mol Microbiol. Januar 2014;91(1):145–57.

29. Anjum MF, Green J, Guest JR. YeiL, the third member of the CRP-FNR family in Escherichia coli. Microbiol Read Engl. Dezember 2000;146 Pt 12:3157–70.

30. Reitzer LJ, Magasanik B. Expression of glnA in Escherichia coli is regulated at tandem promoters. Proc Natl Acad Sci U S A. April 1985;82(7):1979–83.

31. Moskovitz J, Rahman MA, Strassman J, Yancey SO, Kushner SR, Brot N, u. a. Escherichia coli peptide methionine sulfoxide reductase gene: regulation of expression and role in protecting against oxidative damage. J Bacteriol. Februar 1995;177(3):502–7.

32. Ames P, Parkinson JS. Constitutively signaling fragments of Tsr, the Escherichia coli serine chemoreceptor. J Bacteriol. Oktober 1994;176(20):6340–8.

33. Kraxenberger T, Fried L, Behr S, Jung K. First Insights into the Unexplored Two-Component System YehU/YehT in Escherichia coli. J Bacteriol. 15. August 2012;194(16):4272–84.

34. Cai SJ, Inouye M. EnvZ-OmpR Interaction and Osmoregulation in Escherichia coli. J Biol Chem. Juli 2002;277(27):24155–61.

35. Shimada T, Yamazaki K, Ishihama A. Novel regulator PgrR for switch control of peptidoglycan recycling in Escherichia coli. Genes Cells Devoted Mol Cell Mech. Februar 2013;18(2):123–34.

36. Kessler JR, Cobe BL, Richards GR. Stringent Response Regulators Contribute to Recovery from Glucose Phosphate Stress in Escherichia coli. Kelly RM, Herausgeber. Appl Environ Microbiol. 15. Dezember 2017;83(24):e01636-17.

37. Romantsov T, Culham DE, Caplan T, Garner J, Hodges RS, Wood JM. ProP-ProP and ProP-phospholipid interactions determine the subcellular distribution of osmosensing transporter ProP in *E scherichia coli*. Mol Microbiol. Februar 2017;103(3):469–82.

38. Gilchrist CA, Denhardt DT. Escherichia coli rep gene: sequence of the gene, the encoded helicase, and its homology with uvrD. Nucleic Acids Res. 26. Januar 1987;15(2):465–75.

39. Corbett D, Bennett HJ, Askar H, Green J, Roberts IS. SlyA and H-NS Regulate Transcription of the Escherichia coli K5 Capsule Gene Cluster, and Expression of slyA in Escherichia coli Is Temperature-dependent, Positively Autoregulated, and Independent of H-NS. J Biol Chem. November 2007;282(46):33326–35.

40. Yamazaki S, Takei K, Nonaka G. ydjN encodes an S-sulfocysteine transporter required by Escherichia coli for growth on S-sulfocysteine as a sulfur source. FEMS Microbiol Lett. September 2016;363(17):fnw185.

41. Lacey MM, Partridge JD, Green J. Escherichia coli K-12 YfgF is an anaerobic cyclic di-GMP phosphodiesterase with roles in cell surface remodelling and the oxidative stress response. Microbiol Read Engl. September 2010;156(Pt 9):2873–86.

42. Harmon DE, Ruiz C. The Multidrug Efflux Regulator AcrR of Escherichia coli Responds to Exogenous and Endogenous Ligands To Regulate Efflux and Detoxification. mSphere. 21. Dezember 2022;7(6):e0047422.

43. Tanabe M, Szakonyi G, Brown KA, Henderson PJF, Nield J, Byrne B. The multidrug resistance efflux complex, EmrAB from Escherichia coli forms a dimer in vitro. Biochem Biophys Res Commun. 6. März 2009;380(2):338–42.

44. Gatzeva-Topalova PZ, May AP, Sousa MC. Structure and mechanism of ArnA: conformational change implies ordered dehydrogenase mechanism in key enzyme for polymyxin resistance. Struct Lond Engl 1993. Juni 2005;13(6):929–42.

45. Ohyama T, Igarashi K, Kobayashi H. Physiological role of the chaA gene in sodium and calcium circulations at a high pH in Escherichia coli. J Bacteriol. Juli 1994;176(14):4311–5.

46. Gasperotti A, Göing S, Fajardo-Ruiz E, Forné I, Jung K. Function and Regulation of the Pyruvate Transporter CstA in Escherichia coli. Int J Mol Sci. 28. November 2020;21(23):9068.

47. López-Garrido J, Casadesús J. The DamX protein of Escherichia coli and Salmonella enterica. Gut Microbes. Juli 2010;1(4):285–8.

48. Kato A, Ohnishi H, Yamamoto K, Furuta E, Tanabe H, Utsumi R. Transcription of emrKY is regulated by the EvgA-EvgS two-component system in Escherichia coli K-12. Biosci Biotechnol Biochem. Juni 2000;64(6):1203–9.

49. Klemm P, Christiansen G. The fimD gene required for cell surface localization of Escherichia coli type 1 fimbriae. Mol Gen Genet MGG. Januar 1990;220(2):334–8.

50. Bennik MHJ, Pomposiello PJ, Thorne DF, Demple B. Defining a *rob* Regulon in *Escherichia coli* by Using Transposon Mutagenesis. J Bacteriol. Juli 2000;182(13):3794–801.

51. Holdsworth SR, Law CJ. Functional and biochemical characterisation of the Escherichia coli major facilitator superfamily multidrug transporter MdtM. Biochimie. Juni 2012;94(6):1334–46.

52. Tsang MJ, Yakhnina AA, Bernhardt TG. NlpD links cell wall remodeling and outer membrane invagination during cytokinesis in Escherichia coli. PLoS Genet. Juli 2017;13(7):e1006888.

53. Yang J, Hwang JS, Camakaris H, Irawaty W, Ishihama A, Pittard J. Mode of action of the TyrR protein: repression and activation of the tyrP promoter of Escherichia coli. Mol Microbiol. April 2004;52(1):243–56.

54. Brokx SJ, Ellison M, Locke T, Bottorff D, Frost L, Weiner JH. Genome-wide analysis of lipoprotein expression in Escherichia coli MG1655. J Bacteriol. Mai 2004;186(10):3254–8.

55. Kumar S, Doerrler WT. Escherichia coli YqjA, a Member of the Conserved DedA/Tvp38 Membrane Protein Family, Is a Putative Osmosensing Transporter Required for Growth at Alkaline pH. J Bacteriol. Juli 2015;197(14):2292–300.

56. Lee DC, Zheng J, She YM, Jia Z. Structure of Escherichia coli tyrosine kinase Etk reveals a novel activation mechanism. EMBO J. 18. Juni 2008;27(12):1758–66.

57. Liu Y, Li S, Li W, Wang P, Ding P, Li L, u. a. RstA, a two-component response regulator, plays important roles in multiple virulence-associated processes in enterohemorrhagic Escherichia coli O157:H7. Gut Pathog. 2019;11:53.
